# Reallocation of time between device-measured movement behaviours and risk of incident cardiovascular disease

**DOI:** 10.1136/bjsports-2021-104050

**Published:** 2021-09-06

**Authors:** Rosemary Walmsley, Shing Chan, Karl Smith-Byrne, Rema Ramakrishnan, Mark Woodward, Kazem Rahimi, Terence Dwyer, Derrick Bennett, Aiden Doherty

**Affiliations:** 1 Nuffield Department of Population Health, University of Oxford, Oxford, UK; 2 Big Data Institute, Li Ka Shing Centre for Health Information and Discovery, University of Oxford, Oxford, UK; 3 Genomic Epidemiology Group, International Agency for Research on Cancer, Lyon, France; 4 Nuffield Department of Women's and Reproductive Health, University of Oxford, Oxford, UK; 5 Professorial Unit, The George Institute for Global Health, University of New South Wales, Camperdown, New South Wales, Australia; 6 Department of Epidemiology, Johns Hopkins University, Baltimore, Maryland, USA; 7 The George Institute for Global Health, School of Public Health, Imperial College London, London, UK; 8 National Institute of Health Research Oxford Biomedical Research Centre, Oxford University Hospitals NHS Foundation Trust, Oxford, UK; 9 Deep Medicine, Oxford Martin School, University of Oxford, Oxford, UK; 10 Oxford University Hospitals NHS Foundation Trust, Oxford, UK; 11 Heart Group, Clinical Sciences, Murdoch Children’s Research Institute, Melbourne, Victoria, Australia

**Keywords:** cardiovascular diseases, physical activity, sedentary behavior, sleep, methods

## Abstract

**Objective:**

To improve classification of movement behaviours in free-living accelerometer data using machine-learning methods, and to investigate the association between machine-learned movement behaviours and risk of incident cardiovascular disease (CVD) in adults.

**Methods:**

Using free-living data from 152 participants, we developed a machine-learning model to classify movement behaviours (moderate-to-vigorous physical activity behaviours (MVPA), light physical activity behaviours, sedentary behaviour, sleep) in wrist-worn accelerometer data. Participants in UK Biobank, a prospective cohort, were asked to wear an accelerometer for 7 days, and we applied our machine-learning model to classify their movement behaviours. Using compositional data analysis Cox regression, we investigated how reallocating time between movement behaviours was associated with CVD incidence.

**Results:**

In leave-one-participant-out analysis, our machine-learning method classified free-living movement behaviours with mean accuracy 88% (95% CI 87% to 89%) and Cohen’s kappa 0.80 (95% CI 0.79 to 0.82). Among 87 498 UK Biobank participants, there were 4105 incident CVD events. Reallocating time from any behaviour to MVPA, or reallocating time from sedentary behaviour to any behaviour, was associated with lower CVD risk. For an average individual, reallocating 20 min/day to MVPA from all other behaviours proportionally was associated with 9% (95% CI 7% to 10%) lower risk, while reallocating 1 hour/day to sedentary behaviour from all other behaviours proportionally was associated with 5% (95% CI 3% to 7%) higher risk.

**Conclusion:**

Machine-learning methods classified movement behaviours accurately in free-living accelerometer data. Reallocating time from other behaviours to MVPA, and from sedentary behaviour to other behaviours, was associated with lower risk of incident CVD, and should be promoted by interventions and guidelines.

## Introduction

Previous studies have shown low levels of light physical activity^1^ and moderate-to-vigorous physical activity^2^ and high levels of sedentary behaviour^3^ are associated with higher cardiovascular disease (CVD) risk, whereas for sleep a U-shaped association has been found.^4 5^ Due to challenges in measuring and analysing movement behaviours, there is uncertainty about how different combinations of movement behaviours are related to CVD risk.

Recall and reporting bias affect self-reported measurements,^6^ and some behaviours (eg, light physical activity) are hard to capture.^7^ Device-based measurements address these concerns but introduce new challenges. Many studies use hip-worn devices, where mean wear time is typically <15 hours/day and sleep is not measured.^8^ Time in different behaviours has typically been identified using ‘cut-point’ based methods, which use an acceleration threshold to distinguish different intensities of activity.^9^ These methods can only distinguish behaviours based on intensity, and are prone to substantial misclassification,^9–11^ which may materially impact research findings.^12^ As they use only a single metric of intensity to classify the behaviour, there may be substantial unused information in the accelerometer signal. Emerging machine-learning methods could, therefore, allow a wider range of behaviours to be classified accurately: these methods use many features of the data, capture non-linear relationships and can learn relationships from training data beyond what a researcher might hypothesise.^9–11 13^ Most behaviour classification methods have been developed using laboratory-based data.^14^ Using free-living data to develop and validate behaviour classification methods is important to ensure they perform well in practice.^9–11 14 15^

There is uncertainty about how movement behaviours are associated with CVD, as analyses often neglect the fact that people engage in multiple movement behaviours over the course of a day (eg, an individual spending large amounts of time sedentary may also spend small amounts of time in light physical activity).^16^ Further complicating this, a person who increases time spent in one behaviour must compensate by decreasing time spent in others. This means that analyses should address the effect of reallocating time between behaviours.^17^ It also means that movement behaviour data are compositional data, whereby only the relative time spent in different behaviours (and not the absolute time in each behaviour) is informative.^18 19^ An individual cannot increase time spent in light physical activity while holding time in other behaviours fixed. However, they can increase time spent in light physical activity relative to other behaviours, while holding each of those behaviours fixed as a proportion of the remaining day. Methods for analysing compositional data aim to capture and model the relative values of variables.^18 19^ While there is a substantial and rapidly growing evidence base linking the movement behaviour composition to cardiovascular risk factors, evidence on incident disease outcomes is still lacking.^20^ Evidence on how the relative time spent in different behaviours over the whole 24-hour day is associated with disease outcomes is important to inform interventions and guidance aimed at disease prevention.^21 22^ The objective of this study was to investigate the association between device-measured movement behaviours and risk of incident CVD in middle-aged to older-aged adults by:

1. Using free-living ‘ground truth’ data to develop and validate a machine-learning model to classify movement behaviours from wrist-worn accelerometer data.
2. Applying this new model to classify movement behaviours of 87 498 UK Biobank participants who wore an accelerometer.
3. Characterising the association between device-measured movement behaviours and incident CVD, accounting for the compositional nature of movement behaviours.

## Methods

### UK Biobank: a large prospective cohort study

UK Biobank is a population-based prospective cohort study of over 500 000 participants in England, Scotland and Wales (protocol available at https://www.ukbiobank.ac.uk/key-documents/). Between 2006 and 2010, individuals aged 40–69 living within roughly 25 miles of an assessment centre were recruited by letter (all eligible individuals were identified from National Health Service records; response rate 5.5%).^23^ At baseline, participants attended an assessment involving a touchscreen questionnaire, biological sampling, an interview by a trained interviewer and anthropometric measurements.^23^

### Device-based measures of movement behaviours in UK Biobank

Between June 2013 and December 2015, participants with a valid email address (excluding North West region due to participant burden concerns) were invited to wear an accelerometer. A total of 106 053 consenting participants were sent an Axivity AX3 wrist-worn triaxial accelerometer to be worn on the dominant wrist for 7 days.^24^ A readable accelerometer dataset was obtained from 103 683 participants. Initial data processing followed established methods:^24^ participants were excluded if the device could not be calibrated, if more than 1% of readings were ‘clipped’ (fell outside the device’s dynamic range of ±8 *g*) before or after calibration, if they had less than 3 days of data or did not have data in each 1-hour period of the 24 hour cycle (with non-wear time defined as unbroken episodes of at least 60 min during which SD of each axis of acceleration was less than 13.0 m*g*),^24^ or if the average acceleration was implausibly high (>100 m*g*).^24^ Recording interruptions and non-wear time were imputed as the mean behaviour in the corresponding minute of the day on remaining days.

### Classification of movement behaviours using machine-learning methods

#### CAPTURE-24

CAPTURE-24, an accelerometer validation study of 152 adults aged 18–91 recruited by advertisements in Oxford, UK, in 2014–2015,^11^ was used to develop machine-learning classification methods. Participants were asked to wear an Axivity AX3 wrist-worn accelerometer for 24 hours, wear a Vicon Autographer wearable camera while awake during that period, and keep a time use diary.^10^ Using camera images and time use diaries, trained annotators annotated accelerometer data with labels from the Compendium of Physical Activities.^25^ Fine-grained labels were mapped to sleep, sedentary behaviour (eg, sitting working at a computer, watching television), light physical activity behaviours (eg, cooking, self-care) and moderate-to-vigorous physical activity behaviours (MVPA; eg, walking the dog, cycling) (see online supplemental methods and online supplemental table 1). Describing intensity in metabolic equivalent of task (METs), which measure energy expenditure relative to energy expenditure in quiet sitting, these behaviours were defined as:

10.1136/bjsports-2021-104050.supp1 Supplementary data

1. Sleep: non-waking behaviour.
2. Sedentary behaviour: waking behaviour at ≤1.5 METs in a sitting, lying or reclining posture.^26^
3. Light physical activity behaviours: waking behaviour at <3 METs not meeting the sedentary behaviour definition.
4. Moderate-to-vigorous physical activity behaviours: all behaviour at ≥3 METs.^25^

#### Machine-learning for behaviour classification

Using this labelled data from the CAPTURE-24 study, a balanced Random Forest with 100 decision trees was trained to classify the behaviour in 30 s time windows using 50 rotation-invariant time and frequency domain features of the accelerometer signal (online supplemental table 2). As the Random Forest did not use time sequence information, the behaviour sequence was smoothed using a Hidden Markov model. This model treated the Random-Forest-predicted behaviours as ‘emissions’ from an underlying true behaviour sequence, and used the Viterbi algorithm to identify the most likely underlying true sequence given the observed sequence.^27^ Transition probabilities between different behaviours were determined using camera validation data, and probability of the Random Forest predicting each behaviour conditional on the true behaviour was estimated using out-of-bag estimates from the Random Forest. This model structure closely followed our previous work,^10 11^ and more detail is given in online supplemental methods.

Performance was evaluated using leave-one-participant-out cross-validation. Accuracy was used to assess overall agreement between annotator-assigned ‘ground truth’ labels and model-assigned labels, and Cohen’s kappa was used to assess agreement beyond that expected by chance. Precision and recall were used to assess performance on each behaviour, and the confusion matrix was used to show classification patterns for examples of each behaviour. Accuracy, Cohen’s kappa, precision and recall were calculated for each participant individually, and we computed their mean (across participants). To examine how sensitive mean precision and recall were to the results of participants with few examples of a behaviour, the mean was recalculated excluding participants with up to 20 min of a particular behaviour (the online supplemental methods contains more detail on performance evaluation). To assess the performance of our model in the age group of interest, we also calculated mean per-participant accuracy and Cohen’s kappa in participants aged 38 years or older (age group as in a release version of this dataset). We also report a model trained in this age group only in online supplemental methods. Although overall comparison is precluded by the different behaviours classified, we compared precision and recall for MVPA using our model compared with using the standard cut-point of 100 m*g*.^28^ Face validity of the behaviour classification method applied to UK Biobank data was assessed by plotting the behaviour profile of UK Biobank participants across the day.

### Ascertainment of CVD endpoints

UK Biobank has ongoing passive follow-up via linkage to Hospital Episode Statistics (HES; hospital diagnoses from the National Health Service, the provider of almost all UK healthcare) and the UK death register.^23^ CVD was defined as ICD-10 codes I20–25 (ischaemic heart diseases) or I60–69 (cerebrovascular diseases) appearing in HES or on the death register. Participants with CVD prior to accelerometer wear, either HES-recorded or self-reported in the baseline questionnaire, were excluded. Participants who did not experience a CVD outcome were censored at death or the end of the study period as appropriate (28 February 2021 for participants in England and Scotland, 28 February 2018 for participants in Wales).

### Compositional data analysis for movement behaviour data

A compositional data analysis approach was used in the statistical analyses. This approach uses log-ratios (log-transformed ratios between movement behaviours) to describe and adjust for the movement behaviour composition. By using ratios between behaviours, the relative time in different behaviours, rather than the absolute time in any given behaviour, is modelled. For this analysis, we used isometric log-ratio pivot coordinates, a particular set of log-ratios which is widely used in movement behaviour research (see online supplemental methods for more detail).^19 29^

Our results are described by pairwise time reallocation plots, which show the HR associated with reallocating time from one behaviour to another behaviour, and by a plot showing the HR associated with particular reallocations of time between behaviours (eg, reallocating 1 hour/day to sedentary behaviour from all other behaviours proportionally).^30^ All HRs are relative to the mean behaviour composition among included participants, so can be interpreted as showing the outcome associated with reallocating time between behaviours for a hypothetical average individual in our sample. Reallocation results are obtained by using the model to estimate the outcome associated with differences in values of the compositional exposure variables relative to the mean behaviour composition (ie, for different reallocations of time between behaviours; see online supplemental methods for more detail).^12 30 31^

### Statistical analyses

Multivariable-adjusted Cox proportional hazards regression models, with age as the timescale, were used to investigate the association between the movement behaviour composition, modelled using isometric log-ratio pivot coordinates, and incident CVD. A minimally adjusted analysis used age as the timescale and was stratified by sex but had no further adjustment for potential confounders. To address potential sources of confounding, the main analysis used age as the timescale, was stratified by sex and was adjusted for ethnicity (Asian, black, other, white), smoking status (current, ex-smoker or never-smoker), frequency of alcohol consumption (never, <3 times/week, 3+ times/week), fresh fruit and vegetable consumption (<3, 3–4.9, 5–7.9, 8+ servings/day), frequency of red and processed meat consumption (<1, 1–2.9, 3–4.9, 5+ times/week), frequency of oily fish consumption (<1, 1, 2–4, >4 times/week), education (school leaver, further education, higher education) and deprivation (quarter of Townsend Deprivation Index in the study population). As body mass index (BMI) may mediate associations between movement behaviours and CVD, the main analysis was not adjusted for BMI. However, BMI may also act as a confounder for associations between movement behaviours and CVD. Therefore, an additional analysis was further adjusted for BMI. As there was evidence that BMI violated the proportional hazards assumption, this adjustment was performed by stratifying the Cox model by BMI (<25, 25–30, 30+ kg/m^2^).^32^ A further multivariable-adjusted analysis was performed with fatal cardiovascular events as the outcome. Separate analyses were also performed in women and men, and in those aged under 65 vs over 65 at the time of accelerometer wear. All adjustment variables were measured at baseline assessment (online supplemental table 3 gives more details on all variables used in the analysis), and variables were not adjusted for if they were likely mediators of the association between movement behaviours and CVD.

Participants with missing data in any adjustment variable were excluded. The proportional hazards assumption was tested component-wise and globally using the Grambsch-Therneau test with the Kaplan-Meier transformation,^29^ and there was no evidence (at the 5% level) that it was violated in the main analysis. Plots of the Schoenfeld residuals were also examined. Results were reported according to Strengthening the Reporting of Observational Studies in Epidemiology guidelines (STROBE; see online supplemental material),^33^ and all CIs are 95% CIs. Software is described in online supplemental methods.

### Sensitivity analyses

The impact of reverse causality was assessed first by excluding the initial 2 years of follow-up and any events within it. A further analysis additionally excluded participants who self-reported poor health or use of diabetes or CVD-related medications at baseline or who had a prior hospital admission for any condition of the circulatory system (I00–I99 as a primary diagnosis, eg, admission for heart failure or aortic aneurysm).

To investigate unmeasured and residual confounding, we used a negative control outcome of accidents without a plausible mechanistic link to movement behaviours (accidents excluding falls, cycling accidents and intentional self-harm; see online supplemental table 3).^34^ We also used E-values to assess the minimum strength of association that an unmeasured confounder would need with both exposure and outcome to explain away the observed association (see online supplemental methods).^35 36^

Two further sensitivity analyses, addressing the treatment of zero values and comparing with a linear isotemporal substitution approach, are reported in online supplemental methods.

### Patient and public involvement

UK Biobank is a pre-existing resource, with public consultation in its design.^37^ Patients and the public were not involved in the development of the research question or the design of the analysis in this study. Results of studies using UK Biobank data are disseminated to participants via UK Biobank’s website and social media.

## Results

### Movement behaviour classification in the training dataset

Our machine-learning method accurately classified movement behaviours in accelerometer data: when evaluated using leave-one-participant-out cross-validation in 2501 hours of free-living data from the CAPTURE-24 study (online supplemental table 4), mean per-participant accuracy was 88% (95% CI 87% to 89%) and mean per-participant Cohen’s kappa was 0.80 (95% CI 0.79 to 0.82). This was consistent across age groups: in the 72 participants aged 38 years or older, mean per-participant accuracy was 86% (95% CI 85% to 88%) and mean per-participant Cohen’s kappa was 0.79 (95% CI 0.76 to 0.82). Mean per-participant precision and recall for each behaviour show most examples of all behaviours were correctly classified, with highest performance for sleep (online supplemental figure 1). Misclassifications were most common between similar behaviours (table 1). As expected, classification performance was worse on individuals with very few true examples of a behaviour (online supplemental figure 1). While the different behaviours classified preclude an overall comparison, our model identified moderate-to-vigorous physical activity behaviours with substantially higher precision (overall precision 0.75 vs 0.37) and similar recall (overall recall both 0.66) compared with using the standard 100 m*g* cut-point, and had higher face validity in UK Biobank, with median 25 min/day in MVPA according to our model compared with 1.5 hours/day with the standard cutpoint. Overall, the behaviour classification showed high face validity when applied to UK Biobank participants’ data (online supplemental figure 2).

**Table 1:** Minute-wise confusion matrix for machine-learned classification of behaviours in accelerometer data from 152 CAPTURE-24 participants in leave-one-participant-out cross-validation

Model-assigned label	Sleep	Sedentary behaviour	Light physical activity behaviours	Moderate-to-vigorous physical activity behaviours
**‘Ground truth’**				
Sleep	51 347	980	215	0
Sedentary behaviour	2322	53 052	5717	87
Light physical activity behaviours	54	4986	22 217	1533
Moderate-to-vigorous physical activity behaviours	6	158	2434	4978

### Analyses in the UK Biobank

#### Baseline characteristics

**Figure 1:**
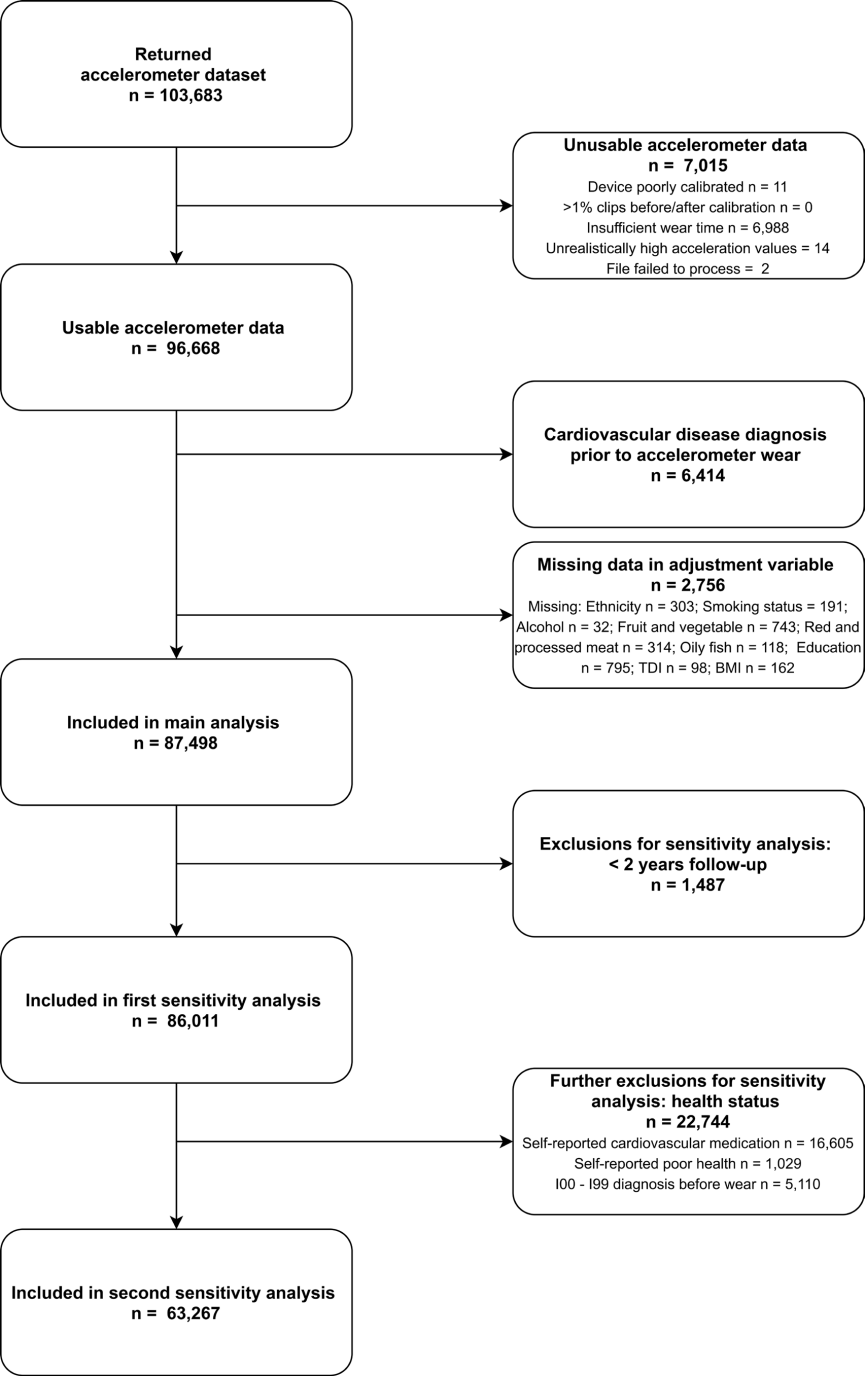
Participant flow diagram for the analysis of movement behaviours and incident cardiovascular disease in UK Biobank participants.BMI, Body Mass Index; TDI, Townsend Deprivation Index.

After excluding participants with poor quality accelerometer data (defined in the Methods section: Device-based measures of movement behaviours in UK Biobank), participants with prevalent ischaemic heart disease or cerebrovascular disease in hospital records or baseline self-report, and participants with missing data, 87 498 UK Biobank participants were included in the Cox regression analysis for incident CVD (figure 1).

**Figure 2:**
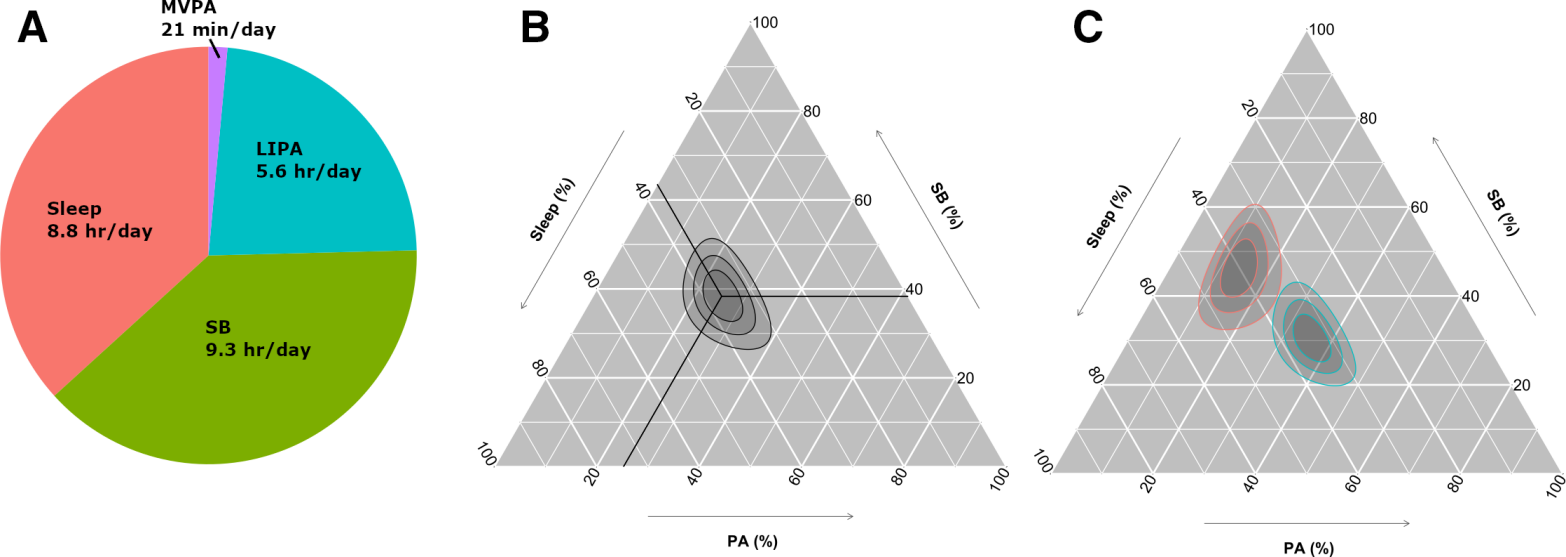
Distribution of movement behaviours in 87 498 UK Biobank participants. (A) Mean movement behaviour composition among UK Biobank participants. (B) Movement behaviours of UK Biobank participants on a ternary plot, showing sleep, sedentary behaviour (SB) and physical activity behaviours (PA; combines light and moderate-to-vigorous physical activity behaviours). The crosshair marks the compositional mean. Concentric rings represent the 25, 50% and 75% prediction regions for the data. The behaviour composition at a point can be found by tracing out (parallel to the white lines and crosshair) from the point to the axes. (C) Ternary plot showing the behaviour distribution of the 5% most active (blue) and 5% least active (red) UK Biobank participants by average acceleration. Concentric rings represent the 25, 50% and 75% prediction regions for each group. LIPA, light physical activity behaviours; MVPA, moderate-to-vigorous physical activity behaviours.

The mean composition of movement behaviours (the daily movement behaviours of a hypothetical average individual) was 8.8 hours/day sleep, 9.3 hours/day sedentary behaviour, 5.6 hours/day light physical activity behaviours and 21 min/day moderate-to-vigorous physical activity behaviours (figure 2). Time in physical activity behaviours and sedentary behaviour varied substantially among participants, while variation in sleep was more limited (figure 2). The least and most active participants by average acceleration differed in all dimensions of behaviour (figure 2).

When considering movement behaviours according to participant characteristics, notable differences included that women had higher levels of light physical activity behaviours than men, and lower sedentary time and MVPA (table 2). Older participants spent less time in MVPA than younger participants (table 2). Participants with higher BMI spent less time in light physical activity behaviours and MVPA than participants with lower BMI, and spent more time sedentary (table 2).

**Table 2:** Movement behaviours of 87 498 UK Biobank participants by participant characteristics

	N (%)*	Sleep† (hr/day)	Sedentary behaviour† (hr/day)	Light physical activity behaviours† (hr/day)	Moderate-to-vigorous physical activity behaviours† (min/day)
Overall	87 498 (100)	8.6 (7.9–9.3)	9.2 (8.0–10.3)	5.5 (4.5–6.7)	25 (12–44)
Age, years					
40–49	7767 (9)	8.5 (7.8–9.1)	9.4 (8.1–10.6)	5.4 (4.3–6.6)	30 (16–50)
50–59	26 081 (30)	8.5 (7.8–9.2)	9.3 (8.1–10.5)	5.4 (4.4–6.7)	28 (14–47)
60–69	38 774 (44)	8.6 (8.0–9.3)	9.0 (7.9–10.2)	5.6 (4.6–6.7)	25 (12–43)
70–79	14 876 (17)	8.6 (7.9–9.4)	9.2 (8.1–10.3)	5.5 (4.5–6.7)	20 (9–36)
Sex					
Female	50 882 (58)	8.6 (8.0–9.3)	8.9 (7.8–10.0)	5.8 (4.8–7.0)	22 (10–38)
Male	36 616 (42)	8.4 (7.8–9.2)	9.6 (8.4–10.8)	5.1 (4.1–6.2)	31 (16–52)
Ethnicity					
Asian	756 (1)	8.4 (7.7–9.3)	9.3 (7.9–10.5)	5.6 (4.5–6.9)	19 (9–35)
Black	701 (1)	8.2 (7.3–9.0)	9.4 (8.0–10.6)	5.9 (4.8–7.2)	21 (10–35)
Other	1151 (1)	8.4 (7.6–9.1)	9.2 (8.0–10.5)	5.7 (4.5–7.0)	26 (13–44)
White	84 890 (97)	8.6 (7.9–9.3)	9.2 (8.0–10.3)	5.5 (4.5–6.7)	25 (12–44)
Smoking status					
Never smoker	50 888 (58)	8.6 (7.9–9.3)	9.2 (8.0–10.3)	5.5 (4.5–6.7)	26 (13–45)
Ex-smoker	30 717 (35)	8.5 (7.9–9.3)	9.2 (8.0–10.3)	5.6 (4.5–6.7)	25 (12–44)
Current smoker	5893 (7)	8.6 (7.9–9.3)	9.4 (8.2–10.6)	5.4 (4.3–6.6)	21 (9–39)
Alcohol consumption					
Never drinker	4745 (5)	8.6 (7.9–9.4)	9.1 (7.8–10.3)	5.6 (4.5–6.9)	20 (9–39)
<3 times per week	39 760 (45)	8.6 (7.9–9.3)	9.2 (8.0–10.3)	5.5 (4.5–6.7)	23 (11–41)
3+ times per week	42 993 (49)	8.5 (7.9–9.2)	9.2 (8.0–10.4)	5.5 (4.5–6.7)	28 (14–47)
Fruit and vegetable consumption					
<3 servings/day	3595 (4)	8.6 (7.8–9.4)	9.7 (8.4–11.0)	5.0 (3.9–6.3)	21 (9–39)
3–4.9 servings/day	14 293 (16)	8.6 (7.9–9.3)	9.4 (8.2–10.6)	5.3 (4.3–6.5)	24 (12–42)
5–7.9 servings/day	36 991 (42)	8.6 (7.9–9.3)	9.2 (8.0–10.3)	5.5 (4.5–6.7)	26 (13–44)
8+ servings/day	32 619 (37)	8.5 (7.9–9.2)	9.0 (7.8–10.1)	5.7 (4.7–6.9)	26 (13–45)
Townsend deprivation index					
Least deprived (< −3.8)	21 913 (25)	8.6 (7.9–9.3)	9.1 (7.9–10.3)	5.6 (4.6–6.7)	24 (12–43)
Second least deprived (−3.8 to −2.5)	21 839 (25)	8.6 (7.9–9.3)	9.1 (8.0–10.3)	5.6 (4.5–6.7)	24 (12–43)
Second most deprived (−2.5 to −0.2)	21 872 (25)	8.6 (7.9–9.3)	9.2 (8.0–10.3)	5.6 (4.5–6.7)	25 (12–44)
Most deprived (> −0.2)	21 874 (25)	8.5 (7.8–9.2)	9.3 (8.1–10.5)	5.4 (4.3–6.6)	27 (13–47)
Education					
School leaver	19 535 (22)	8.7 (8.0–9.5)	8.9 (7.7–10.1)	5.7 (4.7–6.9)	20 (9–36)
Further education	29 061 (33)	8.6 (7.9–9.3)	9.1 (7.9–10.3)	5.6 (4.6–6.8)	23 (11–41)
Higher education	38 902 (44)	8.5 (7.8–9.1)	9.4 (8.2–10.5)	5.4 (4.4–6.5)	30 (16–50)
BMI					
Underweight (<18.5 kg/m^2^)	511 (1)	8.5 (7.9–9.2)	8.5 (7.2–9.6)	6.3 (4.9–7.3)	34 (19–55)
Normal weight (18.5–24.9 kg/m^2^)	35 043 (40)	8.6 (8.0–9.2)	8.8 (7.7–10.0)	5.8 (4.7–6.9)	30 (16–50)
Overweight (25.0–29.9 kg/m^2^)	35 783 (41)	8.5 (7.9–9.3)	9.3 (8.1–10.4)	5.5 (4.4–6.6)	25 (12–43)
Obese (30+ kg/m^2^)	16 161 (18)	8.5 (7.8–9.3)	9.8 (8.5–10.9)	5.1 (4.1–6.3)	16 (7–31)

Movement behaviours are given as median (IQR).

*Percentages may not sum to 100% due to rounding.

†Presented as median (IQR).

BMI, body mass index.

#### Associations with incident CVD

**Figure 3:**
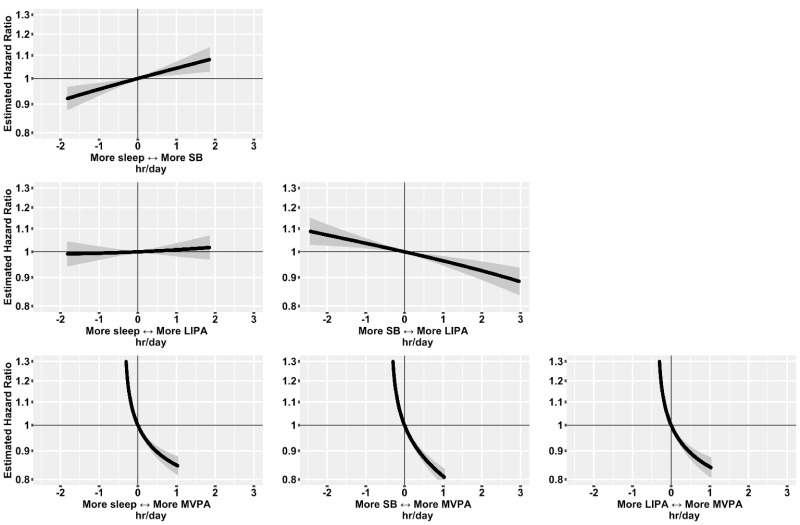
HRs for incident cardiovascular disease associated with balance between movement behaviours in 87 498 UK Biobank participants.Model based on 4105 events in 87 498 participants. All relative to the mean behaviour composition (8.8 hours/day sleep, 9.3 hours/day sedentary behaviour (SB), 5.6 hours/day light physical activity behaviours (LIPA), 0.35 hours/day (21 min/day) moderate-to-vigorous physical activity behaviours (MVPA)). Model used age as the timescale, was stratified by sex and was additionally adjusted for ethnicity, smoking status, alcohol consumption, fresh fruit and vegetable consumption, red and processed meat consumption, oily fish consumption, deprivation and education. 95% CIs shown.

Over 524 919 person years of follow-up (median 6.2 years, maximum 7.7 years), there were 4105 incident CVD events. Reallocating time from sedentary behaviour to light physical activity behaviours was associated with a lower risk of CVD (figure 3): for an average individual in this sample, the HR associated with reallocating 1 hour/day from light physical activity behaviours to sedentary behaviour was 1.04 (95% CI 1.02 to 1.06), while the HR associated with reallocating 1 hour/day from sedentary behaviour to light physical activity behaviours was 0.96 (95% CI 0.95 to 0.98). Reallocating time from sedentary behaviour to MVPA was associated with more pronounced lower risk of CVD (figure 3): for an average individual, the HR associated with reallocating 15 min/day from MVPA to sedentary behaviour was 1.19 (95% CI 1.15 to 1.22), while the HR associated with reallocating 15 min/day from sedentary behaviour to MVPA was 0.92 (95% CI 0.91 to 0.94). Reallocating time from light physical activity behaviours or sleep to MVPA, and reallocating time from sedentary behaviour to sleep were also associated with a lower risk of CVD, while reallocating time from sleep to LIPA was not associated with CVD risk (figure 3).

**Figure 4:**
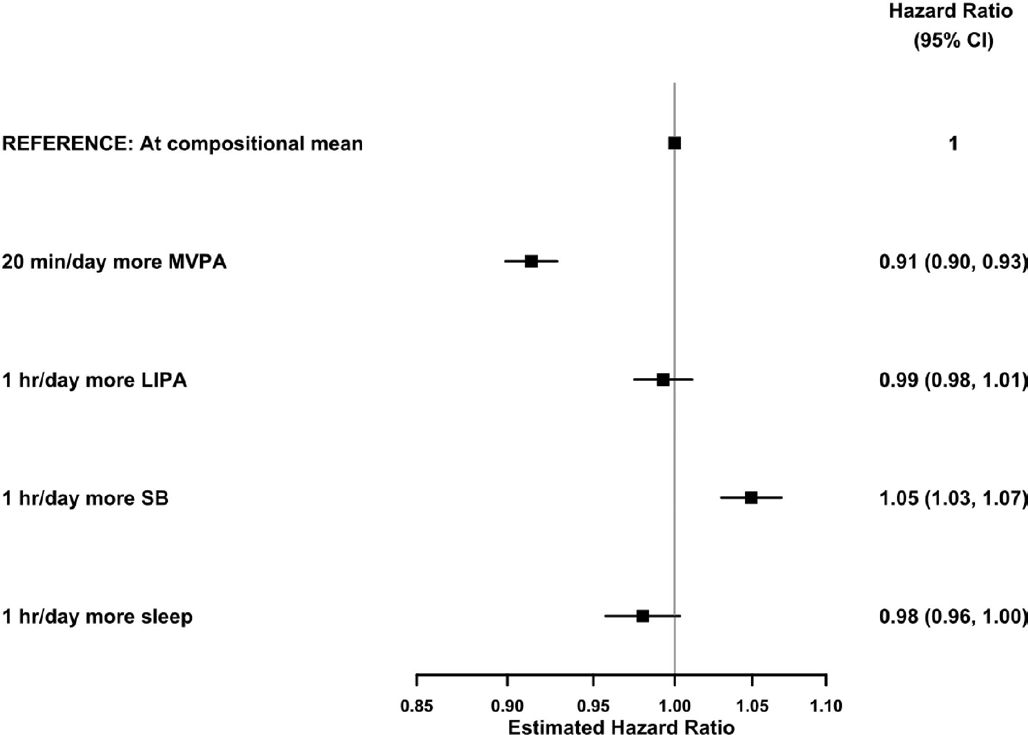
HRs for incident cardiovascular disease associated with reallocating time to named behaviour, from all other behaviours proportionally, in 87 498 UK Biobank participants.Model based on 4105 events in 87 498 participants. All relative to the mean behaviour composition (8.8 hours/day sleep, 9.3 hours/day sedentary behaviour (SB), 5.6 hours/day light physical activity behaviours (LIPA), 0.35 hours/day (21 min/day) moderate-to-vigorous physical activity behaviours (MVPA)) and more time in named behaviour reallocated from all other behaviours proportionally. Model used age as the timescale, was stratified by sex and was additionally adjusted for ethnicity, smoking status, alcohol consumption, fresh fruit and vegetable consumption, red and processed meat consumption, oily fish consumption, deprivation and education. 95% CIs shown.

We found that, for an average individual in this sample, reallocating 20 min/day to MVPA from all other behaviours proportionally was associated with 9% (95% CI 7% to 10%) lower risk of CVD (figure 4; 28% of the study population exceeded this level of MVPA). Reallocating 1 hour/day to sedentary behaviour, from all other behaviours proportionally, was associated with 5% (95% CI 3% to 7%) higher risk of CVD (figure 4; 26% of the study population exceeded this level of sedentary behaviour).

Reallocating 1 hour/day to light physical activity behaviours, from sleep, sedentary behaviour and MVPA proportionally and reallocating 1 hour/day to sleep, from sedentary behaviour, light physical activity behaviours and MVPA proportionally, showed more modest and non-significant associations with lower risk of CVD (1% (95% CI −1% to 2%) and 2% (95% CI 0% to 4%), respectively; figure 4).

Results from the multivariable-adjusted model were only slightly attenuated compared with those from a minimally adjusted model (online supplemental figure 3, online supplemental table 6). Further adjustment for BMI, using a model stratified by BMI category, resulted in some attenuation of the association between movement behaviours and incident CVD (online supplemental figure 4, online supplemental table 6). For example, the 9% (95% CI 7% to 10%) lower risk of CVD relative to at the compositional mean associated with reallocating 20 min/day to MVPA was reduced to a 7% (95% CI 6% to 9%) lower risk after stratification by BMI. Associations for fatal cardiovascular events were similar to those for all cardiovascular events, with reallocating time from sedentary behaviour to light physical activity behaviours and sleep appearing more strongly associated with fatal events (online supplemental figure 5, online supplemental table 6). Results for women and men separately were similar, with some associations appearing stronger for women (online supplemental figure 6, online supplemental table 6). Results for participants aged under and over 65 separately were similar, with some associations appearing stronger for older adults (online supplemental figure 7, online supplemental table 6).

Removing the first 2 years of follow-up attenuated all associations only minimally (online supplemental figure 8, online supplemental table 6). Further restricting to a healthy subgroup, associations for reallocating time into MVPA remained broadly similar, but associations for reallocating time from sedentary behaviour to light physical activity behaviours and to sleep were substantially attenuated (online supplemental figure 8, online supplemental table 6).

Analyses suggested residual and unmeasured confounding had a modest impact on the main findings. Specifically, some movement behaviours were associated with the negative control outcome, suggesting a small impact of residual confounding (online supplemental figure 9, online supplemental table 6). The E-values indicated that a substantial degree of unmeasured confounding would be required to reduce the observed associations to the null for MVPA and sedentary behaviour reallocated from other behaviours proportionally (online supplemental figure 10). For example, the E-value of 1.42 (for reallocating 20 min/day to MVPA, from all other behaviours) shows an unmeasured confounder would need to be associated with at least a 1.42-fold increase in risk for both exposure and outcome to explain away the observed association.

## Discussion

Using free-living ‘ground truth’ data, we showed that machine-learning methods were able to accurately classify movement behaviours in wrist-worn accelerometer data. By applying these methods, we were able to derive device-based measurements of movement behaviours in 87 498 UK Biobank participants. Using compositional data analysis Cox regression, we studied how the allocation of time between behaviours was associated with incident CVD events over a >6-year follow-up period. We found that reallocating time to MVPA from sleep, sedentary behaviour or light physical activity behaviours or reallocating time from sedentary behaviour to light physical activity behaviours or sleep was associated with lower risk of CVD. Per minute, the most pronounced differences in risk were seen for MVPA. BMI explained a modest proportion of the association between movement behaviours and incident CVD.

Our epidemiological findings extend previously reported results by showing how reallocating time between behaviours is associated with cardiovascular risk (after adjustment for other behaviours). The results of this study are consistent with our previous results, which showed a dose–response association across quartiles of device-measured moderate physical activity for cardiovascular events in the UK Biobank (with 42% lower risk in the highest quartile for moderate physical activity compared with the lowest).^38^ Notably, the current study extends those previous results by measuring and adjusting for behaviours throughout the 24-hour day using a compositional data analysis approach. These results are also consistent with results from a study of community-dwelling older women in the USA, which found 69% higher risk of incident CVD in the highest quartile for device-measured sedentary behaviour compared with the lowest,^39^ and 22% lower risk of incident CVD in the highest quartile for light physical activity compared with the lowest.^1^ Again, these studies only partially adjusted for other behaviours within the 24-hour day.^1^ A recent pooled analysis of data from six cohort studies investigated the association between movement behaviours and all-cause mortality using a compositional data analysis approach and found associations broadly similar to those reported here.^12^ However, they noted measurement challenges, including lack of device-measured sleep time in many studies and inaccurate classification using cut-points in wrist-worn accelerometer data, that hampered interpretation of some results.^12^ The behaviour classification methods developed in this study support interpretable epidemiological analyses. For example, we were able to study device-measured sleep as part of the 24-hour day, and found suggestive results, including that reallocating time from sedentary behaviour to sleep was associated with a lower risk of incident CVD. However, in light of remaining challenges in validating sleep measurement and in studying sleep epidemiologically (eg, a fuller treatment may consider factors beyond duration, including sleep quality), these results are best considered as hypothesis generating.

The performance of our behaviour classification model represents an improvement on previously reported machine-learning approaches in free-living data (Cohen’s kappa 0.80 vs 0.68), likely due to careful curation of the behaviour classes in labelled data by two reviewers.^11^ Our approach also performed better than traditional ‘cut-point’ approaches. While some characteristics of the CAPTURE-24 sample differ from UK Biobank, it is a large, varied dataset, and consistent performance of our methods across age groups suggests our methods are relatively robust. We encourage researchers to conduct studies similar to CAPTURE-24 embedded within prospective cohorts with accelerometer data in the future, and where possible to collect data on relevant participant characteristics. Although comparable with UK estimates from other sources,^40^ sleep measurements should be interpreted cautiously: ‘ground truth’ labels for sleep came from a time use diary, which may identify time in bed rather than physiological sleep. In the future, sleep measurements require validation using polysomnography.

### Strengths

This study has several strengths, notably including the use of device-based measurements to characterise movement behaviours in a large, comprehensive prospective study. Compared with self-reported measurements of behaviour, device-based measurements are at reduced risk of recall and reporting bias,^6^ and they can capture behaviours such as light physical activity well.^7^ The use of a wrist-worn device with a full 24-hour wear protocol (with high compliance) allowed the full day of behaviours to be measured.^24^ The use of free-living data with ‘ground truth’ behaviour labels to develop and validate behaviour classification methods ensures they perform well in real-world settings. All methods used in this study are open-source and available for use in other wrist-worn accelerometer datasets. A major strength of the analysis in this study is the appropriate modelling of 24 hour behaviours using a compositional data analysis approach.^18 19^

### Limitations

An important limitation of any observational study is the possibility of reverse causality bias.^41^ After removing the first 2 years of follow-up, associations were only slightly attenuated. However, further restricting analyses to a healthy subgroup attenuated the associations for reallocating time from sedentary behaviour to light physical activity behaviours and sleep. Associations for reallocating time to MVPA were attenuated slightly or not at all. Residual confounding also remains possible, although sensitivity analyses using a negative control outcome and E-values suggested its impact is likely to be modest. While results are presented for reallocations of time between behaviours, these are derived statistically across participants: each participant had a single measurement, so within-participant changes cannot be addressed directly. Validation of the machine-learning methods on another independent dataset would help to further understand their robustness.^13^ Finally, UK Biobank is not representative of the UK population^23^ (eg, low socioeconomic status individuals are under-represented compared with the national population^42^), though a previous study showed exposure–outcome associations found in UK Biobank were similar to results in more representative samples.^43^

**What are the findings?**

- Emerging methods, including machine-learning for behaviour classification and statistical methods addressing the compositional nature of movement behaviours, can enhance epidemiological studies and lead to new health insights.
- Machine-learning methods enabled accurate classification of movement behaviours from free-living wrist-worn device data (accuracy 88%, kappa 0.80).
- Reallocating time to moderate-to-vigorous physical activity behaviours from light physical activity behaviours, sedentary behaviour or sleep was associated with lower risk of incident cardiovascular disease over >6 years of follow-up.
- Reallocating time from sedentary behaviour to other behaviours was also associated with lower risk of incident cardiovascular disease.

**How might it impact on clinical practice in the future?**

- Machine-learning methods for behaviour classification may be used to accurately classify movement behaviours from wrist-worn device data in free-living environments.
- Our findings support existing public health guidance on reallocating time to moderate-to-vigorous physical activity from other behaviours and reallocating time from sedentary behaviour to light physical activity for population-based cardiovascular disease prevention.

### Conclusions

The use of machine-learning and compositional data analysis methods can enhance prospective cohort studies that collect wearable device data, leading to new health insights. The results of this study support the framing of current guidelines and interventions around increasing time spent in MVPA, and reallocating time from sedentary behaviour to light physical activity behaviours where that is infeasible.^44–46^

[Bibr R44]
