# Supplementary material for: Reallocation of time between device-measured movement behaviours and risk of incident cardiovascular disease
Source: Br J Sports Med. 2021 Sep 6;56(18):1008–17. doi: 10.1136/bjsports-2021-104050 (PMC9484395; doi:10.1136/bjsports-2021-104050)
Supplement: Supplementary data [file bjsports-2021-104050supp001.pdf]

BMJ Publishing Group Limited (BMJ) disclaims all liability and responsibility arising from any reliance placed on this supplemental material which has been supplied by the author(s)

Supplementary Material

Supplementary material for **Reallocation of time between device-measured movement behaviours and risk of incident cardiovascular disease**

Rosemary Walmsley MMathPhil, Shing Chan PhD, Karl Smith-Byrne DPhil, Rema Ramakrishnan PhD, Mark Woodward PhD, Kazem Rahimi FRCP, Terence Dwyer MD, Derrick Bennett PhD, Aiden Doherty PhD

Walmsley R, *et al*. *Br J Sports Med* 2022; 56:1008–1017. doi: 10.1136/bjsports-2021-104050

Contents

Supplementary Methods 4
‘Ground truth’ labelling of movement behaviours in image data 4
Machine-learning methods 5
Machine-learning methods: Features 5
Machine-learning methods: Random Forest models 5
Machine-learning methods: Hidden Markov models 5
Machine-learning methods: Evaluation 5
Machine-learning methods: model in participants aged 38 years or older 6
A Compositional Data Analysis approach to movement behaviour data 7
Log-ratio transformation 7
Interpreting isometric log-ratio pivot coordinates 7
Zero values 8
Software 9
Sensitivity analyses: further details on E-values 10
Sensitivity analyses: linear isotemporal substitution 11
References 12
Supplementary Tables and Figures 14
Supplementary Table 1: Assignment of fine-grained camera image annotations from the Compendium of Physical Activities to broad movement behaviour classes. 14
Supplementary Table 2: Features of accelerometry signal used for behaviour classification. 22
Supplementary Table 3: Definition of variables from UK Biobank data included in statistical models. 24
Supplementary Table 4: Characteristics of CAPTURE-24 participants. 28
Supplementary Figure 1: Participant-wise mean (a) precision and (b) recall for classification of behaviours from accelerometer data calculated in Leave-One-Participant-Out Cross-Validation (with 95% confidence interval for the mean). The x-axis gives the minimum required recorded annotator-labelled time in the behaviour for inclusion in the calculation. For precision, participants with no model-labelled time in the behaviour were also excluded as precision is undefined in this case. 29
Supplementary Figure 2: Probability of being in sleep, sedentary behaviour (SB), light physical activity behaviours (LIPA) and moderate-to-vigorous physical activity behaviours (MVPA) among 87,498 UK Biobank participants according to machine-learned behaviour classification by hour of the day. 30
Supplementary Table 5: Coefficient of first isometric log-ratio pivot coordinate[a] for each movement behaviour estimated using a multivariable-adjusted Compositional Data Analysis Cox regression model. 31
Supplementary Table 6: Coefficient of first isometric log-ratio pivot coordinate for each movement behaviour estimated using all Compositional Data Analysis Cox regression models.[a] 32
Supplementary Figure 3: Hazard Ratios for cardiovascular disease for all behaviour pairs estimated using multivariable-adjusted (blue) and minimally adjusted (red) Cox regression models.[a] 33
Supplementary Figure 4: Hazard Ratios for incident cardiovascular disease estimated using a multivariable-adjusted Cox regression model before (blue) and after (red) stratification by BMI.[a] 34
Supplementary Figure 5: Hazard Ratios for all (blue) and fatal (red) incident cardiovascular disease for all behaviour pairs estimated using a multivariable-adjusted Cox regression model.[a] 35
Supplementary Figure 6: Hazard Ratios for incident cardiovascular disease for all behaviour pairs estimated using a multivariable-adjusted Cox regression model for women (blue) and men (red).[a] 36
Supplementary Figure 7: Hazard Ratios for incident cardiovascular disease for all behaviour pairs estimated using a multivariable-adjusted Cox regression model for participants aged under 65 (blue) and participants aged over 65 (red).[a] 37

Supplementary Figure 8: Hazard Ratios for incident cardiovascular disease for all behaviour pairs estimated using a multivariable-adjusted Cox regression model (blue), after removing the first two years of follow-up (red) and after additionally restricting to a healthy subgroup (green).[a] 38

Supplementary Figure 9: Hazard Ratios for cardiovascular disease (blue) and for non-activity-related accidents (red) for all behaviour pairs estimated using a multivariable-adjusted Cox regression model.[a] 39

Supplementary Figure 10: Hazard ratios and corresponding E-values for incident cardiovascular disease associated with reallocating time to named behaviour, from all other behaviours proportionally, in 87,498 UK Biobank participants.[a] 40

Supplementary Figure 11: An example of a decision tree to classify time windows using average acceleration vector magnitude (avm) and the 75th percentile of acceleration vector magnitude (75thp). 41

Supplementary Figure 12: The structure of a Hidden Markov Model. 42

Supplementary Figure 13: Hazard Ratios for incident cardiovascular disease for all behaviour pairs estimated using a multivariable-adjusted Cox regression model for all participants (blue) and in a sensitivity analysis excluding individuals with a zero value in any behaviour (red).[a] 43

Supplementary Figure 14: Hazard Ratios for incident cardiovascular disease estimated using a multivariable-adjusted Compositional Data Analysis Cox regression model (blue) and using a multivariable-adjusted linear isotemporal substitution Cox regression model (red).[a] 44

STROBE Statement—Checklist of items that should be included in reports of cohort studies 45

Supplementary Methods

‘Ground truth’ labelling of movement behaviours in image data

As described in the main text, to provide the ‘ground truth’ labels for machine-learning based behaviour classification, fine-grained behaviour annotations of image data were mapped to sleep, sedentary behaviour, light physical activity behaviours and moderate-to-vigorous physical activity behaviours based on the definition in the main text. In practice, this involved the following steps (the final mapping is given in **Supplementary Table 1**):

1. The fine-grained annotation for sleeping was assigned to sleep.
2. Behaviours at 3 or more METs (Metabolic Equivalent of Task, where 1 MET is energy expenditure in quiet sitting), as described in Compendium of Physical Activities [1], were assigned to moderate-to-vigorous physical activity behaviours.
3. For waking behaviours at <3 METs, if the fine-grained annotation indicated a sitting, lying or reclining posture, the behaviour was assigned to sedentary behaviour.
4. Waking behaviours at <3 METs not assigned to sedentary behaviour were assigned to light physical activity behaviours.
5. All labels were reviewed by two reviewers. Where reviewers agreed the fine-grained annotation was typically used by annotators for behaviours in a different category to the label given, the fine-grained annotation was recoded. This review was performed prior to model training, and no changes were made after results were obtained.

Walmsley R, *et al. Br J Sports Med* 2022; 56:1008–1017. doi: 10.1136/bjsports-2021-104050

Machine-learning methods

As described in the main text, a Random Forest (RF) with 100 decision trees was developed to classify 30-second time windows as sleep, sedentary behaviour, light physical activity behaviours or moderate-to-vigorous physical activity behaviours using the time and frequency domain features outlined in **Supplementary Table 2**. A Hidden Markov model (HMM) was then employed to use time sequence information to improve on the RF-assigned label sequence. As described in the main text, models were trained using labelled data from the CAPTURE-24 study, in which participants wore wearable cameras and kept time use diaries alongside wearing an accelerometer.

Machine-learning methods: Features

Time windows of acceleration were classified using a list of features (variables) based on features used in the study of Willetts et al (this included time and frequency domain features e.g. mean and kurtosis of the acceleration vector magnitude, and power at different frequencies from the Fast Fourier Transform of acceleration vector magnitude) [2]. In the present study, only rotation-invariant features were used (see **Supplementary Table 2**). This addressed concerns about risk of overfitting and possible time trends in the data driven by sensitivity to device orientation within the wrist strap (orientation became more standardised over 2013-2015).

Machine-learning methods: Random Forest models

Random Forests are based on decision trees. Decision trees assign class labels based on splits of the data using feature value thresholds (as shown in the example in **Supplementary Figure 11**). They can be trained using the Classification and Regression Tree (CART) algorithm [3].

In a RF, many decision trees are used. When training the trees (using the CART algorithm), randomness is introduced by (i) training each tree on a set of data points picked randomly (with replacement) and (ii) at each split node, restricting the choice of splitting feature to a randomly picked subset of features.

To classify a data point using an RF, it is classified by each decision tree. Because they are trained on different subsets of data and use different features, different trees in the RF may classify data points differently. The overall classification given by the RF is the class that is assigned to the data point by the largest number of trees. This approach, whereby multiple randomly-differing instances are used in order to reduce variance on the output, is the technique of bootstrap-aggregating or ‘bagging’ [4].

For this application, a balanced RF was used. The fact some behaviours are much more common than others in the labelled data (e.g. sleep is much more common than moderate-to-vigorous physical activity behaviours) can cause a standard RF, which is trained by picking N examples at random with replacement, to favour assigning common labels at the expense of less common behaviours [2]. Using the balanced RF, if there were n_{rare} examples of the rarest behaviour, n_{rare} examples of each behaviour were picked with replacement to train each tree.

Machine-learning methods: Hidden Markov models

In a HMM, there is a sequence of unobserved hidden states, which is assumed to have the Markov property (i.e. future states only depend on past states through present states). This sequence is governed by transition probabilities, which determine the probability of transitioning between each pair of states. There is a sequence of observed states, which depend probabilistically on the sequence of hidden states (described as ‘emissions’ from the sequence of hidden states; **Supplementary Figure 12**).

Here, the hidden states were the true behaviours, and the emissions were the RF-assigned labels. The Viterbi algorithm, the standard approach to this problem, was used to estimate the most likely true behaviour sequence given the observed sequence of RF-assigned labels [5]. Applying the Viterbi algorithm required estimates of:

1. **Transition probabilities between hidden states:** Transition probabilities between behaviour pairs were estimated using the proportions of transitions that occurred between each behaviour pair in the labelled data.
2. **Emission probabilities of observed states from hidden states:** To estimate emission probabilities, time windows were first classified using out-of-bag predictions from the RF i.e. trees were used to classify data points on which they were not trained. This mimics use on unseen data, without requiring additional data. Emission probabilities were then estimated using the proportions of different pairs of true behaviour and RF out-of-bag estimate.

By using this HMM to estimate the most likely true behaviour sequence given the RF-assigned labels, a more plausible sequence of states was obtained. The HMM re-labelled behaviours which formed unrealistic sequences and were likely to be attributable to misclassification (e.g. short periods of moderate-to-vigorous physical activity behaviours during sleep time). Therefore, compared to the unadjusted RF-assigned labels, the labels after using the HMM gave improved measures of the behaviours of interest for subsequent epidemiological analyses.

Machine-learning methods: Evaluation

All metrics were calculated in Leave-One-Participant-Out Cross-Validation.

Leave-One-Participant-Out Cross-Validation involves, for each participant, a model trained on all other participants’ data (i.e. with this participant’s data left-out). The trained model is then used to label the left-out participant’s data and evaluation metrics are calculated. This is repeated for all participants, and metrics are aggregated or calculated across all participants.

Leave-One-Participant-Out Cross-Validation allows evaluation of the performance of the models on data not used in training, while retaining the maximal amount of data for use in training these models. Moreover, all of the data can then be used to train the final model used for classification.

For model performance, the following evaluation metrics were used:

1. We reported mean per-participant accuracy across all behaviours. This is a simple, intuitive metric of model performance, describing the proportion of 30-second time windows that were correctly classified. Using mean per-participant accuracy, rather than aggregate accuracy over all data, prevents the result being dominated by performance on a few participants with larger amounts of data (important as there may be inter-individual differences in classification performance).
2. We reported mean per-participant Cohen's kappa across all behaviours. This is a metric of interrater reliability. It evaluates how much higher the agreement between two raters (here, annotator-assigned 'ground truth' label and model-assigned label) is than that which would be achieved by chance, given the proportions in each class. It is preferable to accuracy, as it takes into account the proportions in each class (in particular, in data where some classes are dominant, a classifier assigning solely to the dominant classes can achieve high accuracy but not high Cohen's kappa).
3. We reported mean per-participant precision and recall for each behaviour. Precision for a given behaviour is the proportion of examples labelled by the model as that behaviour which are 'true' examples of that behaviour. Recall for a given behaviour is the proportion of 'true' examples of that behaviour labelled as that behaviour. Again, taking the mean across participants prevents performance being dominated by performance on participants with larger amounts of data. However, it also upweights the contribution of individuals with very small amounts of data for a given behaviour. Therefore, precision and recall were additionally calculated after excluding participants with up to 20 minutes in the behaviour.

Taken together, these metrics help to understand the validity of the model as a method to derive measures of movement behaviours for subsequent epidemiological analyses. After applying the model to derive measures of movement behaviours for UK Biobank participants, face validity was assessed by plotting behaviour profiles over the day.

Machine-learning methods: model in participants aged 38 years or older

We also carried out the above steps using only data from participants aged 38 years or older i.e. nearer to the age group represented in the UK Biobank sample. The age group 38+ years was used as this corresponds to the information available in a release version of this dataset.

In Leave-One-Participant-Out analysis, the mean per-participant accuracy was 86% (84, 88) and the mean per-participant Cohen's kappa was 0.79 (0.76, 0.81).

Given the results reported in the main text, showing that the model trained on all participants performed well when restricted to the age group of interest, we used the model trained on all participants for the main classification.

A Compositional Data Analysis approach to movement behaviour data

Log-ratio transformation

A Compositional Data Analysis approach is a set of methods for working with compositional data, based on the use of log-transformed ratios to describe the data [6–8]. Ratios between behaviours are used to describe compositional data as they capture the relative values of the different behaviours. Log-transforming ratios ensures the relationships and distances between different compositions are well-described: using log-transformed ratios is equivalent to working with compositional data in a 'natural' space for it, with operations which map compositions to genuine compositions and an appropriate distance metric [9,10]. For statistical purposes, log-transformed ratios are also typically more conveniently distributed than ratios [11].

While many different sets of log-transformed ratios can be used, isometric log-ratio pivot coordinates are widely used in movement behaviour research [12] and were used in this study. They were calculated as follows:

$$coordinate_1 = \sqrt{\frac{3}{4}}\ln\left(\frac{\text{sleep}}{\sqrt[3]{\text{SB}\times\text{LIPA}\times\text{MVPA}}}\right) = \sqrt{\frac{1}{12}}\ln\left(\frac{sleep}{SB}\right) + \sqrt{\frac{1}{12}}\ln\left(\frac{sleep}{LIPA}\right) + \sqrt{\frac{1}{12}}\ln\left(\frac{sleep}{MVPA}\right)$$

$$coordinate_2 = \sqrt{\frac{2}{3}}\ln\left(\frac{\text{SB}}{\sqrt[2]{\text{LIPA}\times\text{MVPA}}}\right) = \sqrt{\frac{1}{6}}\ln\left(\frac{SB}{LIPA}\right) + \sqrt{\frac{1}{6}}\ln\left(\frac{SB}{MVPA}\right)$$

$$coordinate_3 = \sqrt{\frac{1}{2}}\ln\left(\frac{\text{LIPA}}{\text{MVPA}}\right)$$

Interpreting isometric log-ratio pivot coordinates

As the coefficients in the model relate to the isometric log-ratio pivot coordinates, rather than the raw behaviour variables, interpreting them is not straightforward.

The first coordinate describes the balance between sleep and all other behaviours. Therefore, the coefficient of the first coordinate can be interpreted as the effect of reallocating time to sleep from all other behaviours proportionally i.e. if the coefficient of the first coordinate is greater than 0 (its exponent is greater than 1) reallocating time to sleep from all other behaviours proportionally is associated with higher risk of cardiovascular disease. However, the second and third coordinates are harder to interpret analogously. Therefore, to interpret individually the effect of reallocating time to each behaviour from all others proportionally, and following standard methods in movement behaviour research, one model per behaviour was produced (with different first coordinate). This approach was used to present the model parameters in **Supplementary Table 5** and **Supplementary Table 6** (note that, in consequence, they do not parametrise a single model).

However, even using this approach, the magnitudes of the coefficients are hard to interpret. Therefore, as described in the main text, and following established methods, model estimates of the hazard ratio at different compositions relative to the mean behaviour composition were reported e.g. using pairwise time reallocation plots.

In detail, suppose we have a Cox regression model in the isometric log-ratio pivot coordinates laid out above:

$$\ln\frac{h(t)}{h_0(t)} = \beta_1(coordinate_1) + \beta_2(coordinate_2) + \beta_3(coordinate_3) + \sum_{i=1}^{k}\gamma_i(covariate_i)$$

and that the value of the coordinates at the mean behaviour composition is $(m\_coordinate_1, m\_coordinate_2, m\_coordinate_3)$. [This notation is not misleading: the value of the coordinates at the compositional mean behaviour composition is also the mean of the coordinate values.].

Then, we consider a new behaviour composition, which corresponds to isometric log-ratio pivot coordinate values $(coordinate_1, coordinate_2, coordinate_3)$. In particular, when considering a pairwise time reallocation plot we use would use a new composition where time in two behaviours remained at its value in the mean behaviour composition, a value was subtracted from time in one of the remaining behaviours, and that value added to the time in the other behaviour. The log hazard ratio, now relative to the mean behaviour composition, is then calculated as:

$$\ln(HR) = \beta_1(coordinate_1 - m\_coordinate_1) + \beta_2(coordinate_2 - m\_coordinate_2) + \beta_3(coordinate_3 - m\_coordinate_3)$$

The standard error on this can be calculated using the variance-covariance matrix of the coefficients $\boldsymbol{\beta} = (\beta_1, \beta_2, \beta_3)$, denoted V. Writing $\boldsymbol{x} = (coordinate_1 - m\_coordinate_1, coordinate_2 - m\_coordinate_2, coordinate_3 - m\_coordinate_3)^T$, then

$$\text{SE} = \sqrt{\boldsymbol{x}^T V \boldsymbol{x}}$$

This can be used to calculate the HR and an (approximate) 95% CI as:

Walmsley R, *et al. Br J Sports Med* 2022; 56:1008–1017. doi: 10.1136/bjsports-2021-104050

$$\exp(\ln(HR))\ (\exp(\ln(HR) - 1.96SE)\,, \exp(\ln(HR) + 1.96SE)).$$

While here we follow an exposition similar to that of Dumuid et al in the context of linear regression [13] and Chastin et al [14], the approach is mathematically equivalent to that of McGregor et al.[15]

To ensure results were plotted within the range supported by the data, times plotted were truncated at the 5th and 95th centile for the behaviour in the pair for which this range was narrower.

Zero values

All participants recorded time in sleep, sedentary behaviour and light physical activity behaviours, but 1% of participants recorded no time in moderate-to-vigorous physical activity behaviours. As zero values cannot be incorporated directly into the coordinates above, different approaches to work with them have been developed. The appropriate method depends on the source of the zero values:

1. 'Rounded' zeroes relate to measurement precision: even where no time in a given behaviour was observed, had the wear time been long enough, or the time resolution of the measurement short enough, some time in the behaviour would be expected. If data contains rounded zeroes, they can be imputed as small positive values [16].
2. 'True' zeroes occur where no matter the precision of the measurement, no time in that behaviour would be observed. For example, this may occur in movement behaviour research if someone is physically unable to take part in certain behaviours. If data contains true zeroes, participants with a true zero in a particular behaviour should be excluded from the main analysis and analysed separately.

We followed established methods in movement behaviour research by considering zero values to be 'rounded' and imputing them using the log-ratio expectation maximization algorithm from the 'zCompositions' R package [8,15–17]. We used the smallest observed value in the data as the detection limit (0.0001 on the unitless scale, corresponding to 0.14 min/day). Sensitivity of results to the method of treating zero values (imputation or exclusion) under the Compositional Data Analysis approach was examined by performing an analysis restricted to participants with non-zero values in all behaviour variables. This did not materially impact the results (**Supplementary Figure 13**).

Software

Development of the machine-learning models and processing of accelerometer data used Python 3.6.6, with the 'biobankAccelerometerAnalysis' tool[2,18,19] for preparing accelerometer data and training machine-learning models.

Data preparation was performed in Python 3.6.6 and R 4.0.5, and used the 'ukb_download_and_prep_template' tool [20] for preparing covariate and outcome data.

Statistical analysis was performed in R 4.0.5[21] with 'zCompositions', 'survival', 'forestplot', 'EValue', 'plyr', 'data.table', 'rlist', 'ggtern', 'ggplot2', and 'gtools'[16,22–32]. The R package 'epicoda' was developed for this analysis[33].

Code is available at github.com/activityMonitoring/manuscript_ml_behaviours_cvd_2021. For further directions, please contact Aiden Doherty.

Sensitivity analyses: further details on E-values

As described in the main text, E-values were reported alongside hazard ratios. The E-value for the estimate quantifies the minimum strength of association that an unmeasured confounder would need with both exposure and outcome to explain away the observed association. The E-value for the 95% confidence interval quantifies the minimum strength of association an unmeasured confounder would need with both exposure and outcome to reduce the interval to overlap the null [27,28]. As the exposure is continuous, in both cases the risk ratio would apply to hypothetical groups with either the specified behaviour composition or the reference (the mean behaviour composition) [28].

Sensitivity analyses: linear isotemporal substitution

For comparability with previous literature, a sensitivity analysis using a linear isotemporal substitution approach was conducted.

Under a linear isotemporal substitution approach, all but one of the movement behaviours are included in the model (so the included variables are not perfectly multicollinear). [In this study, as non-wear time was imputed all subjects had the same wear time. Therefore, a total time variable was not included, meaning the approach may be more properly called 'leave-one-out regression' than true linear isotemporal substitution [8].] Associations are modelled as linear (rather than linear in the log-ratios, as under a Compositional Data Analysis approach). The coefficient of each behaviour can be interpreted in terms of replacing time in the left-out behaviour with time in that behaviour. Linear isotemporal substitution has been widely used in movement behaviour epidemiology [34], but has been criticised for not addressing the fact movement behaviour data only conveys relative information [8].

While there were some differences in shape of the associations observed (due to the different assumptions), results using this approach were broadly similar to the results of the main analysis using Compositional Data Analysis (**Supplementary Figure 14**).

Walmsley R, *et al. Br J Sports Med* 2022; 56:1008–1017. doi: 10.1136/bjsports-2021-104050

References

1 Ainsworth BE, Haskell WL, Herrmann SD, *et al.* 2011 compendium of physical activities: A second update of codes and MET values. *Med Sci Sports Exerc* 2011;**43**:1575–81. doi:10.1249/MSS.0b013e31821ece12

2 Willetts M, Hollowell S, Aslett L, *et al.* Statistical machine learning of sleep and physical activity phenotypes from sensor data in 96,220 UK Biobank participants. *Sci Rep* 2018;**8**:0–10. doi:10.1038/s41598-018-26174-1

3 Breiman L, Friedman JH, Olshen RA, *et al. Classification and regression trees*. 1984. doi:10.1201/9781315139470

4 Breiman L. Bagging predictors. *Mach Learn* 1996;**24**:123–40. doi:10.1007/bf00058655

5 Forney GD. The Viterbi Algorithm. *Proc IEEE* 1973;**61**:268–78. doi:10.1109/PROC.1973.9030

6 Pawlowsky-Glahn V, Egozcue JJ, Tolosana-Delgado R. Lecture notes on Compositional Data Analysis. 2011;:1–100. doi:10.13140/RG.2.1.1296.2728

7 Filzmoser P, Hron K, Templ M. *Applied Compositional Data Analysis*. 2018. doi:10.1007/978-3-319-96422-5

8 Dumuid D, Pedišić Ž, Palarea-Albaladejo J, *et al.* Compositional Data Analysis in Time-Use Epidemiology : What , Why , How. *Int J Environ Res Public Health* 2020;**17**:2220. doi:10.3390/ijerph17072220

9 Aitchison J, Egozcue JJ. Compositional data analysis: Where are we and where should we be heading? *Math Geol* Published Online First: 2005. doi:10.1007/s11004-005-7383-7

10 Egozcue JJ, Pawlowsky-Glahn V, Mateu-Figueras G, *et al.* Isometric Logratio Transformations for Compositional Data Analysis. *Math Geol* 2003;**35**:279–300. doi:10.1023/A:1023818214614

11 Aitchison J. The statistical analysis of compositional data. *Stat Anal Compos Data* 1982;**44**:139–77. doi:10.1007/978-94-009-4109-0

12 Dumuid D, Stanford TE, Martin-Fernández J-AA, *et al.* Compositional data analysis for physical activity, sedentary time and sleep research. *Stat Methods Med Res* 2017;**27**:096228021771083. doi:10.1177/0962280217710835

13 Dumuid D, Pedišić Ž, Stanford TE, *et al.* The compositional isotemporal substitution model: A method for estimating changes in a health outcome for reallocation of time between sleep, physical activity and sedentary behaviour. *Stat Methods Med Res* 2017;**28**:846–57. doi:10.1177/0962280217737805

14 Chastin S, McGregor D, Palarea-Albaladejo J, *et al.* Joint association between accelerometry-measured daily combination of time spent in physical activity, sedentary behaviour and sleep and all-cause mortality: a pooled analysis of six prospective cohorts using compositional analysis. *Br J Sports Med* 2021;:bjsports-2020-102345. doi:10.1136/bjsports-2020-102345

15 McGregor DE, Palarea-Albaladejo J, Dall PMP, *et al.* Cox regression survival analysis with compositional covariates: Application to modelling mortality risk from 24-h physical activity patterns. *Stat Methods Med Res* 2019;**29**:1447–65. doi:10.1177/0962280219864125

16 Palarea-Albaladejo J, Martín-Fernández JA. ZCompositions - R package for multivariate imputation of left-censored data under a compositional approach. Chemom. Intell. Lab. Syst. 2015;**143**:85–96. doi:10.1016/j.chemolab.2015.02.019

17 Rasmussen CL, Palarea-Albaladejo J, Johansson MS, *et al.* Zero problems with compositional data of physical behaviors: a comparison of three zero replacement methods. *Int J Behav Nutr Phys Act* 2020.

18 Doherty A, Jackson D, Hammerla N, *et al.* Large scale population assessment of physical activity using wrist worn accelerometers: The UK biobank study. *PLoS One* 2017;**12**:1–14. doi:10.1371/journal.pone.0169649

19 Doherty A, Smith-Byrne K, Ferreira T, *et al.* GWAS identifies 14 loci for device-measured physical activity and sleep duration. *Nat Commun* 2018;**9**. doi:10.1093/acrefore/9780190236557.013.204

20 Walmsley R, Chan S, Doherty A. ukb_download_and_prep_template. 2020.https://github.com/activityMonitoring/ukb_download_and_prep_template

21 R Core Team. R: A Language and Environment for Statistical Computing. *Vienna, Austria* Published Online First: 2019.https://www.r-project.org/

22 Therneau TM. A Package for Survival Analysis in R. 2021.https://cran.r-project.org/package=survival

23 Wickham H. *ggplot2: Elegant Graphics for Data Analysis*. New York: : Springer-Verlag 2016. https://ggplot2.tidyverse.org

24 Warnes GR, Bolker B, Lumley T. gtools: Various R Programming Tools. *R Packag version 381* 2018.

25 Terry M. Therneau, Patricia M. Grambsch. *Modeling Survival Data: Extending the Cox Model*. New York: : Springer 2000.

26 Max Gordon and Thomas Lumley. forestplot: Advanced Forest Plot Using ‘grid’ Graphics. R package version 1.9. 2019.https://cran.r-project.org/package=forestplot

27 Van Der Weele TJ, Ding P. Sensitivity analysis in observational research: Introducing the E-Value. *Ann Intern Med* 2017;**167**:268–74. doi:10.7326/M16-2607

28 Mathur MB, Ding P, Riddell CA, *et al.* Web Site and R Package for Computing E-values. *Epidemiology* 2018;**29**:e45. doi:10.1097/EDE.0000000000000864

29 Wickham H. The Split-Apply-Combine Strategy for Data Analysis. *J Stat Softw* 2011;**40**:1–29.http://www.jstatsoft.org/v40/i01/

30 Dowle M, Srinivasan A. data.table: Extension of `data.frame`. 2021.https://cran.r-project.org/package=data.table

31 Ren K. rlist: A Toolbox for Non-Tabular Data Manipulation. 2016.https://cran.r-project.org/package=rlist

32 Hamilton NE, Ferry M. Ggtern: Ternary diagrams using ggplot2. *J Stat Softw* 2018;**87**:1–17. doi:10.18637/jss.v087.c03

33 Walmsley R. epicoda. 2020.github.com/activityMonitoring/epicoda

34 Mekary RA, Ding EL. Isotemporal substitution as the gold standard model for physical activity epidemiology: Why it is the most appropriate for activity time research. *Int J Environ Res Public Health* 2019;**16**:11–3. doi:10.3390/ijerph16050797

Supplementary Tables and Figures

Supplementary Table 1: Assignment of fine-grained camera image annotations from the Compendium of Physical Activities to broad movement behaviour classes.

**Sleep**
7030 sleeping
**Sedentary behaviour**
occupation;office and administrative support;11580 office/computer work general
occupation;office and administrative support;11580 office wok/computer work general
home activity;miscellaneous;sitting;9060 sitting/lying reading or without observable/identifiable activities
leisure;miscellaneous;sitting;0010 sitting / lying dialysis
transportation;private transportation;16010 driving automobile or light truck (not a semi)
home activity;miscellaneous;sitting;7010 sitting/lying and watching television with TV on as the primary activity
home activity;miscellaneous;sitting;11580 office/computer work general
home activity;miscellaneous;sitting;9055 sitting/lying talking in person/using a mobile phone/smartphone/tablet or talking on the phone/computer (skype chatting)
home activity;miscellaneous;sitting;11580 office work such as writing and typing (with or without eating at the same time)
home activity;eating;13030 eating sitting alone or with someone
home activity;miscellaneous;sitting;9030 sitting desk entertainment/hobby (with or without eating at the same time)
home activity;miscellaneous;sitting;5080 sitting non-desk work (with or without eating at the same time)
occupation;interruption;sitting;11585 sitting meeting/talking to colleagues with or without eating
leisure;miscellaneous;sitting;9060 (generic) sitting/lying reading or without observable/identifiable activities
occupation;interruption;11585 sitting meeting/talking to colleages with or without eating
home activity;leisure;activities for maintenance of a household;miscellaneous;9100 retreat/family reunion activities involving sitting eating relaxing talking with more than one person
home activity;miscellaneous;sitting;9060 sitting/lying reading or without observable activities
leisure;eating;social;13030 eating sitting indoor/outdoor
home activity;leisure;activties for maintenance of a household;miscellaneous;9100 retreat/family reunion activities involving sitting eating relaxing talking with more than one person
occupation;public admin/education/health;education;9065 students/attending seminars or talks
home activity;miscellaneous;sitting;9030 sitting desk work (with or without eating at the same time)
occupation;interruption;sitting;9060 sitting without observable/identifiable activities

Walmsley R, *et al. Br J Sports Med* 2022; 56:1008–1017. doi: 10.1136/bjsports-2021-104050

leisure;miscellaneous;sitting;9055 sitting talking to person/using the phone
transportation;private transportation;16015 riding in a car or truck
leisure;eating;13030 eating sitting indoor/outdoor
occupation;interruption;13030 eating sitting
home activity;miscellaneous;sitting;9045 sitting playing traditional video game computer game
home activity;miscellaneous;sitting;9030 sitting desk work (with or without eating at the same time)
occupation;interruption;sitting;13030 eating sitting
home activity;leisure;individual activities;9075 sitting arts and crafts carving/wood weaving/spinning wool
home activity;household chores;washing/ironing/mending clothes;5080 knitting sewing sitting
occupation;interruption;sitting;9055 sitting using a mobile phone/smartphone/tablet or talking on the phone/computer (skype meeting etc.)
leisure;miscellaneous;sitting;21000 sitting meeting
leisure;miscellaneous;sitting;21005 (generic) sitting light office writing typing work
occupation;office and administrative support;11580 office work/computer work general
home activity;miscellaneous;sitting;7010 lying and watching television with TV on as the primary activity
leisure;miscellaneous;sitting;9060 (generic) sitting/lying reading or without observable activities
transportation;waiting;7021 sitting
occupation;interruption;11585 sitting meeting/talking to colleagues with or without eating
leisure;religious activities;20000 sitting in church in service attending a ceremony sitting quietly
transportation;private transportation;16030 motor scooter motorcycle
occupation;interruption;9055 sitting using a mobile phone/smartphone/tablet or talking on the phone/computer (skype meeting etc.)
leisure;miscellaneous;21005 (generic) sitting light office writing typing work
home activity;miscellaneous;sitting;7021 sitting without observable actiivties
home activity;leisure;activties for maintenance of a household;with children;5170 sitting playing with child(ren)
home activity;leisure;activities for maintenance of a household;with children;5170 sitting playing with child(ren)
occupation;interruption;9060 (generic) sitting without observable activities
home activity;child/elderly/pet care;child care;5185 child care sitting/kneeling
home activity;miscellaneous;sitting;21010 sitting non-desk work (with or without eating at the same time)
leisure;eating;not-social;13030 eating sitting indoor/outdoor
leisure;miscellaneous;sitting;5080 sitting non-desk work (with or without eating at the same time)
home activity;leisure;activties for maintenance of a household;9100 retreat/family reunion activities involving sitting eating relaxing talking with more than one person

leisure;miscellaneous;sitting;21016 sitting child care only active periods
home activity;leisure;activties for maintenance of a household;5170 sitting playing with child(ren)
home activity;lawn and garden;gardening service;8055 driving tractor
occupation;interruption;9060 (generic) sitting without observable/identifiable activities
home activity;self care;13036 taking medication
leisure;miscellaneous;21000 sitting meeting or talking with others
home activity;self care;13046 having hair or nails done by someone else sitting
home activity;household chores;washing/ironing/mending clothes;5080 knitting sewing wrapping presents sitting
home activity;miscellaneous;sitting;9060 sitting reading or using a mobile phone/smartphone/tablet or talking on the phone/computer (skype chatting)
leisure;miscellaneous;21010 sitting non-desk work (with or without eating at the same time)
occupation;interruption;9060 sitting using a mobile phone/smartphone/tablet or talking on the phone/computer (skype meeting etc.)
leisure;religious activities;20005 sitting in church talking or singing attending a ceremony sitting active
home activity;child/elderly/pet care;child care;5185 child care sitting/kneeling occasional lifting
leisure;miscellaneous;21016 sitting child care only active periods
home activity;miscellaneous;sitting;7021 sitting without observable activities
home activity;leisure;activities for maintenance of a household;with animals;5190 sitting playing with animals active periods
transportation;public transportation;16016 riding in a bus or train
leisure;sports;water activities;18012 boating power passenger
**Light physical activity behaviours**
home activity;household chores;preparing meals/cooking/washing dishes;5035 kitchen activity general cooking/washing/dishes/cleaning up
home activity;miscellaneous;walking;17150 walking household without observable loads
home activity;miscellaneous;walking;5165 (generic) walking non-cleaning task such as closing windows lock door putting away items
leisure;miscellaneous;walking;21070 (generic) walking and occasional standing (no more than two consecutive images)
transportation;walking;17161 walking not as the single means of transports e.g.from house to transports or vice versa/from car to places or vice versa/between transports
leisure;miscellaneous;walking;5060 shopping miscellaneous
occupation;interruption;11791 walking on job in office or lab area
home activity;miscellaneous;standing;9050 standing talking in person/on the phone/computer (skype chatting) or using a mobile phone/smartphone/tablet
home activity;household chores;washing/ironing/mending clothes;5090 folding or hanging clothes/put clothes in or out of washer or dryer/packing suitcase limited walking

Walmsley R, *et al. Br J Sports Med* 2022; 56:1008–1017. doi: 10.1136/bjsports-2021-104050

home activity;miscellaneous;standing;9070 standing reading or without observable/identifiable activities
occupation;interruption;walking;11791 walking on job in office or lab area
occupation;manufacturing;11115 chef
home activity;self care;13040 (generic) self care such as grooming/washing hands/shaving/brushing teeth/putting on make-up not eliminating and bathing (not necessary in the toilet)
occupation;office and administrative support;11600 (generic) standing tasks such as store clerk/libarian/packing boxes/repair heavy parts
leisure;miscellaneous;5060 shopping miscellaneous
home activity;household chores;washing/ironing/mending clothes;5070 ironing
leisure;miscellaneous;standing;9050 standing talking in person/using a phone/smartphone/tablet
home activity;miscellaneous;walking;5147 walking moving away light items (pens/papers/keys not included)
occupation;miscellaneous;11475 (generic) manual labour
occupation;interruption;standing;9050 standing talking in person/using a phone/smartphone/tablet
occupation;personal services;11413 kitchen maid
home activity;household chores;grocery shopping;5060 shopping
home activity;home repair;indoor;6126 home repair miscellaneous
leisure;miscellaneous;standing;9070 standing reading or without observable/identifiable activities
transportation;waiting;7040 standing in a line
home activity;child/elderly/pet care;child care;5185 child care sitting/kneeling occasional lifting
home activity;miscellaneous;standing;9071 (generic) standing miscellaneous
leisure;miscellaneous;standing;9071 (generic) standing miscellaneous
occupation;interruption;standing;9070 standing reading or without observable/identifiable activities
home activity;miscellaneous;standing;9020 standing writing/drawing/painting
home activity;leisure;individual activities;10074 playing musical instruments
occupation;interruption;5041 kitchen activity in the working place
home activity;miscellaneous;walking;17140 using crutches or frame
occupation;interruption;9050 standing talking in persone/using a phone/smartphone/tablet
home activity;leisure;individual activities;9085 standing arts and crafts/sand painting/carving weaving
home activity;miscellaneous;5025 (generic) multiple household tasks all at once including standing/lifting/sitting
home activity;miscellaneous;standing;9070 standing reading or without obvious activities
home activity;household chores;washing/ironing/mending clothes;5095 putting away /gathering clothes involving walking
home activity;lawn and garden;lawn care service;8135 planting potting transplanting seedlings or plants

occupation;interruption;standing;9071 (generic) standing miscellaneous
transportation;walking;9050 standing miscellaneous (talking to others etc.)
occupation;interruption;miscellaneous;5041 kitchen activity in the working place
home activity;household chores;house cleaning;miscellaneous;5100 making bed/changing linens
occupation;interruption;standing;9015 standing scanning documents
home activity;household chores;house cleaning;furniture;5032 dusting or polishing furniture
home activity;eating;13035 eating standing alone or with others
occupation;miscellaneous;11870 working in scene shop theatre actor backstage employee
occupation;office and administrative support;11600 (generic) standing tasks such as store clerk/librarian/packing boxes/repair heavy parts
home activity;miscellaneous;standing;9050 standing talking in person on the phone/computer (skype chatting) or using a mobileo phone/smartphone/tablet
home activity;household chores;house cleaning;floors;5010 cleaning sweeping carpet or floors
leisure;sports;ball games;15090 bowling
home activity;self care;13020 dressing/undressing
occupation;interruption;9070 standing reading or without observable/identifiable activities
leisure;music playing;10074 playing musical instruments
home activity;home repair;indoor;6205 sharpening tools
home activity;home repair;indoor;6124 hammering nails
home activity;child/elderly/pet care;child care;5186 child care standing occasional lifting
occupation;interruption;13035 eating standing
home activity;child/elderly/pet care;pet care;5197 household animal care aside from feeding
leisure;recreation;outdoor;5171 standing playing with child(ren)
occupation;interruption;miscellaneous;13009 toilet break
home activity;household chores;house cleaning;miscellaneous;5148 watering plants
occupation;miscellaneous;11475 (generic) manual or unskilled labour
occupation;interruption;standing;9020 standing writing/drawing/painting
leisure;eating;social;13035 eating standing indoor/outdoor
home activity;household chores;house cleaning;floors;5131 scrubbing floors on hands and knees scrubbing bathroom bathtub
occupation;public admin/education/health;education;09071 teaching standing
home activity;household chores;preparing meals/cooking/washing dishes;5051 serving food/setting table implied walking and standing
home activity;household chores;preparing meals/cooking/washing dishes;5035 cleaning up table after meal implied walking (e.g. leaving from eating table to the kitchen)

leisure;eating;not-social;5060 buying foods or drinks as a takeaway
leisure;miscellaneous;21070 (generic) walking/standing combination indoor
home activity;self care;13009 toilet eliminating or squatting
home activity;self care;13045 hairstyling standing
home activity;lawn and garden;gardening service;8230 watering garden
occupation;interruption;13009 toilet break
leisure;miscellaneous;standing;9070 standing reading or without obvious activities
home activity;leisure;activities for maintenance of a household;miscellaneous;9101 retreat/family reunion activities playing games with more than one person
occupation;interruption;9015 standing scanning documents
home activity;child/elderly/pet care;child care;5183 standing holding child
occupation;interruption;9070 standing reading or without obvious activities
leisure;miscellaneous;standing;9020 standing writing/drawing/painting
leisure;religious activities;20039 walking/standing combination for religious purposes usher
leisure;recreation;indoor;9020 drawing writing painting standing
leisure;miscellaneous;21017 standing child care only active periods
home activity;child/elderly/pet care;pet care;5053 feeding household animals
leisure;eating;13035 eating standing indoor/outdoor
leisure;miscellaneous;9071 (generic) standing miscellaneous indoor or outdoor
leisure;sports;conditioning;2115 upper body exercise arm ergometer
occupation;interruption;standing;9050 standing talking in persone/using a phone/smartphone/tablet
home activity;self care;13000 getting ready for bed standing
leisure;eating;social;5060 buying foods or drinks as a takeaway
home activity;home repair;indoor;5160 standing light effort tasks
transportation;walking;9071 standing miscellaneous (talking to others etc.)
occupation;interruption;standing;13035 eating standing
occupation;interruption;9020 standing writing/drawing/painting
home activity;lawn and garden;gardening service;8220 walking applying fertilizer or seeding a lawn push applicator
leisure;eating;5060 buying foods or drinks as a takeaway
leisure;religious activities;20030 standing talking in church
leisure;eating;not-social;13035 eating standing indoor/outdoor

occupation;public admin/education/health;education;9071 teaching standing<br>home activity;household chores;washing/ironing/mending clothes;5082 sewing with a machine
**Moderate-to-vigorous physical activity behaviours**<br>transportation;private transportation;1010 bicycling<br>transportation;walking;17165 walking the dog<br>occupation;interruption;17133 walking upstairs<br>leisure;miscellaneous;walking;17031 loading /unloading a car implied walking<br>home activity;child/elderly/pet care;child care;5181 walking and carrying child<br>home activity;lawn and garden;gardening service;8050 digging spading filling garden compositing<br>leisure;sports;miscellaneous;17082 hiking or walking at a normal pace through fields and hillsides<br>home activity;lawn and garden;tree and shrub service;8025 clearing light brush thinning garden<br>home activity;lawn and garden;lawn care service;8165 raking lawn<br>occupation;interruption;walking;11795 walking on job and carrying light objects such as boxes or pushing trolleys<br>leisure;sports;water activities;18070 canoeing/rowing<br>home activity;lawn and garden;lawn care service;8080 laying crushed rock<br>leisure;sports;gymnasium and athletics;athletics;12150 running<br>leisure;sports;ball games;15235 football or baseball playing catch<br>home activity;miscellaneous;standing;5146 standing packing/unpacking household items occasional lifting<br>home activity;lawn and garden;gardening service;8245 gardening/picking up fruits vegetables flowers<br>leisure;miscellaneous;walking;17105 pushing a wheelchair non-occupational<br>leisure;sports;ball games;15680 tennis doubles<br>home activity;lawn and garden;gardening service;8192 shovelling dirt or mud<br>leisure;sports;miscellaneous;17082 hiking or walking at a normal pace through fields and hillsides<br>leisure;dancing;3010 ballet modern or jazz general rehearsal or class<br>leisure;sports;conditioning;2060 health club exercise<br>leisure;sports;ball games;15690 tennis singles<br>home activity;lawn and garden;lawn care service;8095 mowing lawn<br>occupation;public admin/education/health;health;11615 nursing patient care

Walmsley R, *et al. Br J Sports Med* 2022; 56:1008–1017. doi: 10.1136/bjsports-2021-104050

occupation;miscellaneous;11615 (generic) standing lifting items continuously with limited walking
leisure;miscellaneous;17031 loading /unloading a car implied walking
leisure;sports;conditioning;2019 bicycling stationary RPM/Spin bike class
home activity;leisure;activties for maintenance of a household;with animals;5192 walking/running playing with animals active periods
home activity;lawn and garden;gardening service;8192 shoveling dirt or mud
home activity;household chores;house cleaning;furniture;5020 cleaning heavy such as car/windows/garage
leisure;sports;conditioning;2048 elliptical trainer
occupation;interruption;11795 walking on job and carrying light objects such as boxes or pushing trolleys
leisure;sports;conditioning;2050 resistance training
leisure;sports;conditioning;2070 rowing stationary ergometer
leisure;recreation;outdoor;5175 walking/running playing with child(ren)
leisure;sports;conditioning;2010 bicycling stationary
leisure;sports;conditioning;2120 water aerobics water calisthenics water exercise
home activity;home repair;outdoor;6020 automobile body work
occupation;agriculture/forestry/fishing;11192 taking care of animals
leisure;miscellaneous;standing;21017 standing child care only active periods
leisure;sports;conditioning;2065 stair-treadmill ergometer general
home activity;household chores;washing/ironing/mending clothes;5092 washing clothes by hand (with or without hanging wash)
leisure;miscellaneous;walking;17133 walking upstairs
transportation;walking;12150 running
occupation;interruption;walking;17070 walking downstairs
home activity;household chores;house cleaning;floors;5140 sweeping garage sidewalk or outside of house
occupation;interruption;walking;17133 walking upstairs
occupation;agriculture/forestry/fishing;11540 shovelling digging ditches
occupation;construction;11050 carrying heavy loads
transportation;walking;17250 walking as the single means to a destination not to work or class
leisure;miscellaneous;walking;17070 descending stairs
home activity;miscellaneous;walking;5121 walking with moving and lifting loads such as bikes and furniture
transportation;walking;17270 walking as the single means to work or class (not from)

Supplementary Table 2: Features of accelerometry signal used for behaviour classification.

Feature	Description
enmoTrunc	Euclidean Norm Minus One truncated below at 0
mean	Mean
sd	Standard Deviation
coefvariation	Coefficient of Variation
median	Median
min	Minimum
max	Maximum
25thp	25th percentile
75thp	75th percentile
autocorr	Autocorrelation
fmax	Frequency of signal with highest power
pmax	Maximal power of signal
fmaxband	Frequency of signal with highest power between 0·3 and 3 Hz
pmaxband	Maximal power of signal between 0·3 and 3 Hz
entropy	Entropy
fft1	Power at 1Hz
fft2	Power at 2Hz
fft3	Power at 3Hz
fft4	Power at 4Hz
fft5	Power at 5Hz
fft6	Power at 6Hz
fft7	Power at 7Hz
fft8	Power at 8Hz
fft9	Power at 9Hz
fft10	Power at 10Hz
fft11	Power at 11Hz
fft12	Power at 12Hz

Feature	Description
MAD	Mean Amplitude Deviation
MPD	Mean Power Deviation
skew	Skew
kurt	Kurtosis
f1	Frequency of signal with highest power between 0·3 and 15 Hz
p1	Maximal power of signal between 0·3 and 15 Hz
f2	Frequency of signal with second highest power between 0.3 and 15 Hz
p2	Second highest power of signal between 0.3 and 15 Hz
f625	Frequency of signal with highest power between 0·6 and 2·5 Hz
p625	Maximal power of signal between 0·6 and 2·5 Hz
totalPower	Total power for frequencies between 0·3 and 15 Hz
vmfft1	Power at 1/30 Hz
vmfft2	Power at 2/30 Hz
vmfft3	Power at 3/30 Hz
vmfft4	Power at 4/30 Hz
vmfft5	Power at 5/30 Hz
vmfft6	Power at 6/30 Hz
vmfft7	Power at 7/30 Hz
vmfft8	Power at 8/30 Hz
vmfft9	Power at 9/30 Hz
vmfft10	Power at 10/30 Hz
vmfft11	Power at 11/30 Hz
vmfft12	Power at 12/30 Hz

Supplementary Table 3: Definition of variables from UK Biobank data included in statistical models.

Characteristic	Source	Notes	UK Biobank field	Coding
OUTCOME				
Age at first cardiovascular disease event	Death Registry, HES.	First appearance of ICD-10 codes I20-25 (ischaemic heart disease) or I60-69 (cerebrovascular disease) in either HES or death register data.	Derived from Category 100093, Category 2000.	
Age at loss-to-follow-up	Death Registry.	Earliest of: date of latest study data available and date of non-cardiovascular disease death.	Derived from Category 100093.	
EXPOSURE				
Sleep	Accelerometry.	Derived using machine-learning methods described in main text.	Derived from 90001.	
Sedentary behaviour	Accelerometry.	Derived using machine-learning methods described in main text.	Derived from 90001.	
Light physical activity behaviours	Accelerometry.	Derived using machine-learning methods described in main text.	Derived from 90001.	
Moderate-to-vigorous physical activity behaviours	Accelerometry.	Derived using machine-learning methods described in main text.	Derived from 90001.	
EXCLUSION VARIABLES – MAIN ANALYSIS				
Prior HES-recorded cardiovascular disease	HES.	Appearance of ICD-10 codes I20-25 (ischaemic heart disease) or I60-69 (cerebrovascular disease) or ICD-9 codes 410 – 414 or 430 – 438 in HES data prior to accelerometer wear.	Derived from Category 2000.	

Walmsley R, *et al. Br J Sports Med* 2022; 56:1008–1017. doi: 10.1136/bjsports-2021-104050

Characteristic	Source	Notes	UK Biobank field	Coding
Prior self-reported cardiovascular disease	Baseline.	Self-reported heart attack or stroke.	Derived from 6150.	
ADJUSTMENT VARIABLES – MAIN ANALYSIS				
Age	Baseline.	Age used as the timescale in Cox regression analysis; participants entered study at the end of accelerometer wear.	Derived from 90011, 34, 52.	
Sex	Baseline.	Used to stratify analysis.	31	Female, Male.
Ethnicity	Baseline.		Derived from 21000.	Asian, Black, Other, White. (Categorised as in Resource 100336; Other includes Mixed, Chinese and Other due to small numbers).
Smoking status	Baseline.		20116	Never smoker, Ex-smoker, Current smoker.
Frequency of alcohol consumption	Baseline.		Derived from 1558.	Never, <3 times/week, 3+ times/week.
Education	Baseline.		Derived from 6138.	School leaver, Further education (education beyond O-Levels/CSEs, excluding college/ university degree) and Higher education (college/university degree).
Townsend Deprivation Index	Baseline.	Townsend Deprivation Index of address at time of UKB baseline assessment.	Derived from 189.	Divided by quartile in the study population.
Daily servings of fresh fruit and vegetables.	Baseline.		Derived from 1289, 1299, 1309.	Less than one coded as 0·5, then sum of 1289, 1299, 1309. Categorised as < 3, 3-4·9, 5-7·9, 8+ servings/ day.

Walmsley R, *et al. Br J Sports Med* 2022; 56:1008–1017. doi: 10.1136/bjsports-2021-104050

Characteristic	Source	Notes	UK Biobank field	Coding
Frequency of red and processed meat consumption.	Baseline.		Derived from 1369, 1379, 1389, 1349.	Less than one coded as 0·5, then sum of 1369, 1379, 1389. Categorised as <1, 1-2·9, 3-4·9, 5+ times/ week.
Frequency of oily fish consumption.	Baseline.		Derived from 1329.	< 1, 1, 2-4, >4 times/week.
ADJUSTMENT VARIABLES – ADDITIONAL				
BMI	Baseline.		Derived from 21001.	For descriptive analyses: Underweight (<18·5kgm^{-2}), Normal weight (18·5 -24·9 kgm^{-2}), Overweight (25 -29·9 kgm^{-2}), Obese (30+ kgm^{-2}). For BMI-stratified analysis, Underweight and Normal weight categories combined.
EXCLUSION VARIABLES – SENSITIVITY ANALYSIS FOR REVERSE CAUSALITY				
Medication for diabetes, cholesterol or blood pressure	Baseline.		Derived from 6177, 6153.	
Self-reported overall health rating	Baseline.		2178	
Prior primary admission for disease of the circulatory system.	HES.	Hospital admission with primary diagnosis of I00-I99 before accelerometer wear.	Derived from Category 2000.	
NEGATIVE CONTROL OUTCOME – SENSITIVITY ANALYSIS FOR RESIDUAL CONFOUNDING				
Age at first accident unrelated to movement behaviour.	Death Registry, HES.	First appearance of ICD-10 codes V00-09, V20-99, W20-W99, X00-X59.	Derived from Category 100093, Category 2000.	
Age at loss-to-follow-up	Death Registry.	Earliest of: date of latest study data available and date of death.	Derived from Category 100093.	

Characteristic	Source	Notes	UK Biobank field	Coding
Prior HES-recorded accident unrelated to movement behaviour.	HES.	First appearance of ICD-10 codes V00-09, V20-99, W20-W99, X00-X59.	Derived from Category 2000.	

Supplementary Table 4: Characteristics of CAPTURE-24 participants.

	n (%)
Overall	152
Age, years	
18-29	39 (26)
30-39	41 (27)
40-49	24 (16)
50-59	21 (14)
60-69	17 (11)
70-79	7 (5)
80-91	2 (1)
Not recorded	1 (1)
Sex	
Female	99 (65)
Male	53 (35)

Walmsley R, *et al. Br J Sports Med* 2022; 56:1008–1017. doi: 10.1136/bjsports-2021-104050

Supplementary Figure 1: Participant-wise mean (a) precision and (b) recall for classification of behaviours from accelerometer data calculated in Leave-One-Participant-Out Cross-Validation (with 95% confidence interval for the mean). The x-axis gives the minimum required recorded annotator-labelled time in the behaviour for inclusion in the calculation. For precision, participants with no model-labelled time in the behaviour were also excluded as precision is undefined in this case.

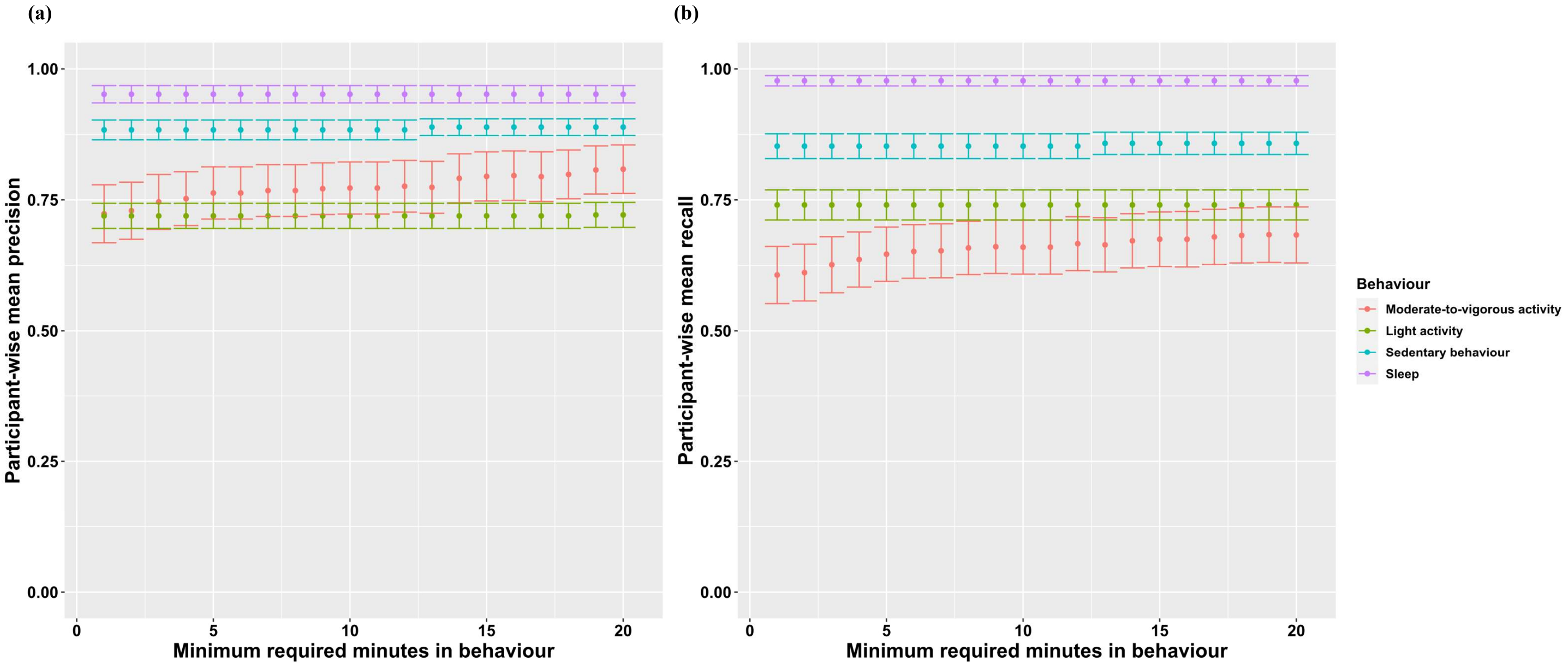

Supplementary Figure 2: Probability of being in sleep, sedentary behaviour (SB), light physical activity behaviours (LIPA) and moderate-to-vigorous physical activity behaviours (MVPA) among 87,498 UK Biobank participants according to machine-learned behaviour classification by hour of the day.

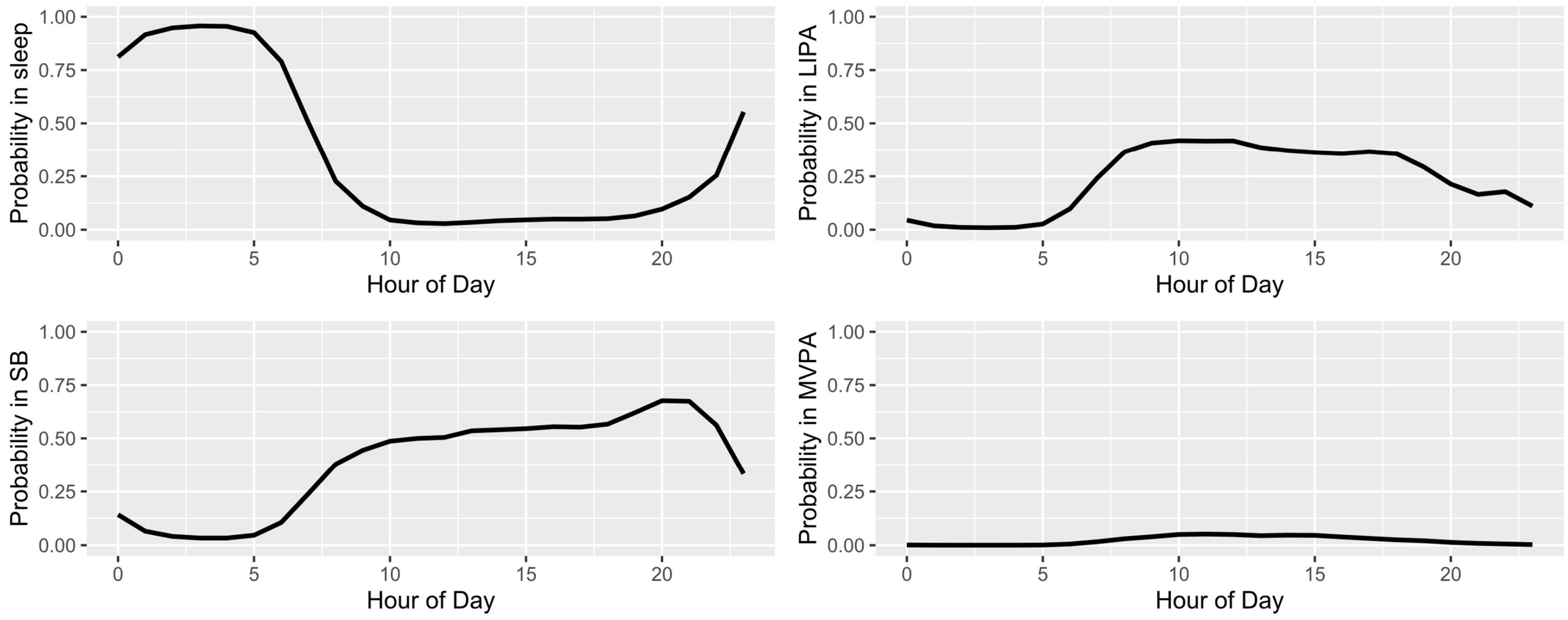

Walmsley R, *et al. Br J Sports Med* 2022; 56:1008–1017. doi: 10.1136/bjsports-2021-104050

Supplementary Table 5: Coefficient of first isometric log-ratio pivot coordinate[a] for each movement behaviour estimated using a multivariable-adjusted Compositional Data Analysis Cox regression model.

Movement behaviour variable	$exp(\hat{\beta})$ (95% CI)
Pivot coordinate: Sleep vs All other behaviours	0·88 (0·75, 1·02)
Pivot coordinate: SB vs All other behaviours	1·38 (1·22, 1·57)
Pivot coordinate: LIPA vs All other behaviours	0·96 (0·88, 1·06)
Pivot coordinate: MVPA vs All other behaviours	0·86 (0·84, 0·88)

[a]See **Supplementary Methods**. Model based on 4,105 events in 87,498 participants. Model used age as the timescale, was stratified by sex and was additionally adjusted for ethnicity, smoking status, alcohol consumption, fresh fruit and vegetable consumption, red and processed meat consumption, oily fish consumption, deprivation and education.

Supplementary Table 6: Coefficient of first isometric log-ratio pivot coordinate for each movement behaviour estimated using all Compositional Data Analysis Cox regression models.[a]

Movement behaviour variable	Main analysis	Minimally adjusted	Additionally stratified by BMI	Fatal events	Women only	Men only
Pivot coordinate: sleep vs All other behaviours	0.88 (0.75, 1.02)	0.90 (0.77, 1.05)	0.90 (0.77, 1.05)	0.79 (0.48, 1.29)	0.78 (0.60, 1.02)	0.93 (0.77, 1.13)
Pivot coordinate: SB vs All other behaviours	1.38 (1.22, 1.57)	1.37 (1.21, 1.56)	1.29 (1.13, 1.46)	2.13 (1.40, 3.22)	1.57 (1.28, 1.94)	1.28 (1.09, 1.50)
Pivot coordinate: LIPA vs All other behaviours	0.96 (0.88, 1.06)	0.96 (0.88, 1.06)	0.98 (0.89, 1.08)	0.71 (0.52, 0.95)	0.95 (0.81, 1.11)	0.97 (0.86, 1.09)
Pivot coordinate: MVPA vs All other behaviours	0.86 (0.84, 0.88)	0.84 (0.81, 0.86)	0.88 (0.85, 0.90)	0.85 (0.78, 0.92)	0.85 (0.82, 0.89)	0.86 (0.83, 0.90)

Movement behaviour variable	Main analysis	Under 65s only	Over 65s only	First 2 years of follow-up removed	Follow-up removed + healthy subgroup	Excluding zero values	Negative control: accidents
Pivot coordinate: sleep vs All other behaviours	0.88 (0.75, 1.02)	0.98 (0.75, 1.28)	0.83 (0.69, 1.00)	0.88 (0.73, 1.06)	1.01 (0.77, 1.31)	0.86 (0.73, 1.01)	0.91 (0.69, 1.20)
Pivot coordinate: SB vs All other behaviours	1.38 (1.22, 1.57)	1.17 (0.95, 1.45)	1.52 (1.30, 1.77)	1.33 (1.15, 1.55)	1.09 (0.89, 1.35)	1.38 (1.21, 1.57)	1.09 (0.87, 1.36)
Pivot coordinate: LIPA vs All other behaviours	0.96 (0.88, 1.06)	1.01 (0.86, 1.18)	0.93 (0.82, 1.04)	0.98 (0.88, 1.10)	1.04 (0.88, 1.21)	0.99 (0.89, 1.09)	1.10 (0.93, 1.30)
Pivot coordinate: MVPA vs All other behaviours	0.86 (0.84, 0.88)	0.86 (0.82, 0.91)	0.86 (0.83, 0.89)	0.87 (0.84, 0.90)	0.88 (0.83, 0.92)	0.86 (0.83, 0.89)	0.92 (0.87, 0.97)

[a]See Methods, Results and Supplementary Figures 3-13 for more details of models. All columns report $exp(\hat{\beta})$ (95% CI).

Supplementary Figure 3: Hazard Ratios for cardiovascular disease for all behaviour pairs estimated using multivariable-adjusted (blue) and minimally adjusted (red) Cox regression models.[a]

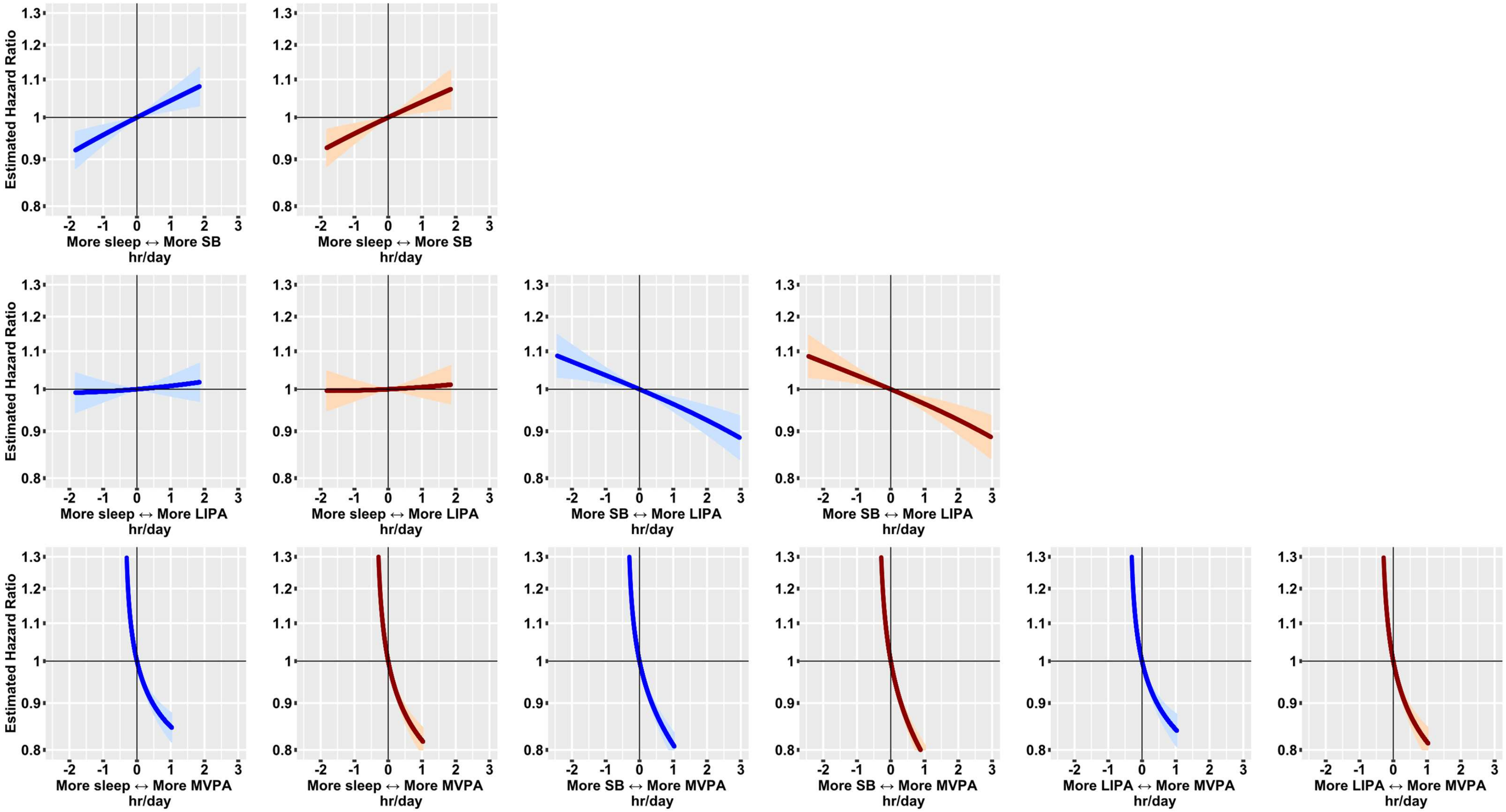

[a]Model based on 4,105 events in 87,498 participants. All relative to the mean behaviour composition: 8·8 hours/day sleep, 9·3 hours/day sedentary behaviour, 5·6 hours/day light physical activity behaviours, 0·35 hours/day (21 minutes/day) moderate-to-vigorous physical activity behaviours. Multivariable-adjusted model used age as the timescale, was stratified by sex and was additionally adjusted for ethnicity, smoking status, alcohol consumption, fresh fruit and vegetable consumption, red and processed meat consumption, oily fish consumption, deprivation and education. Minimally-adjusted model used age as the timescale and was stratified by sex.

Walmsley R, *et al. Br J Sports Med* 2022; 56:1008–1017. doi: 10.1136/bjsports-2021-104050

Supplementary Figure 4: Hazard Ratios for incident cardiovascular disease estimated using a multivariable-adjusted Cox regression model before (blue) and after (red) stratification by BMI.[a]

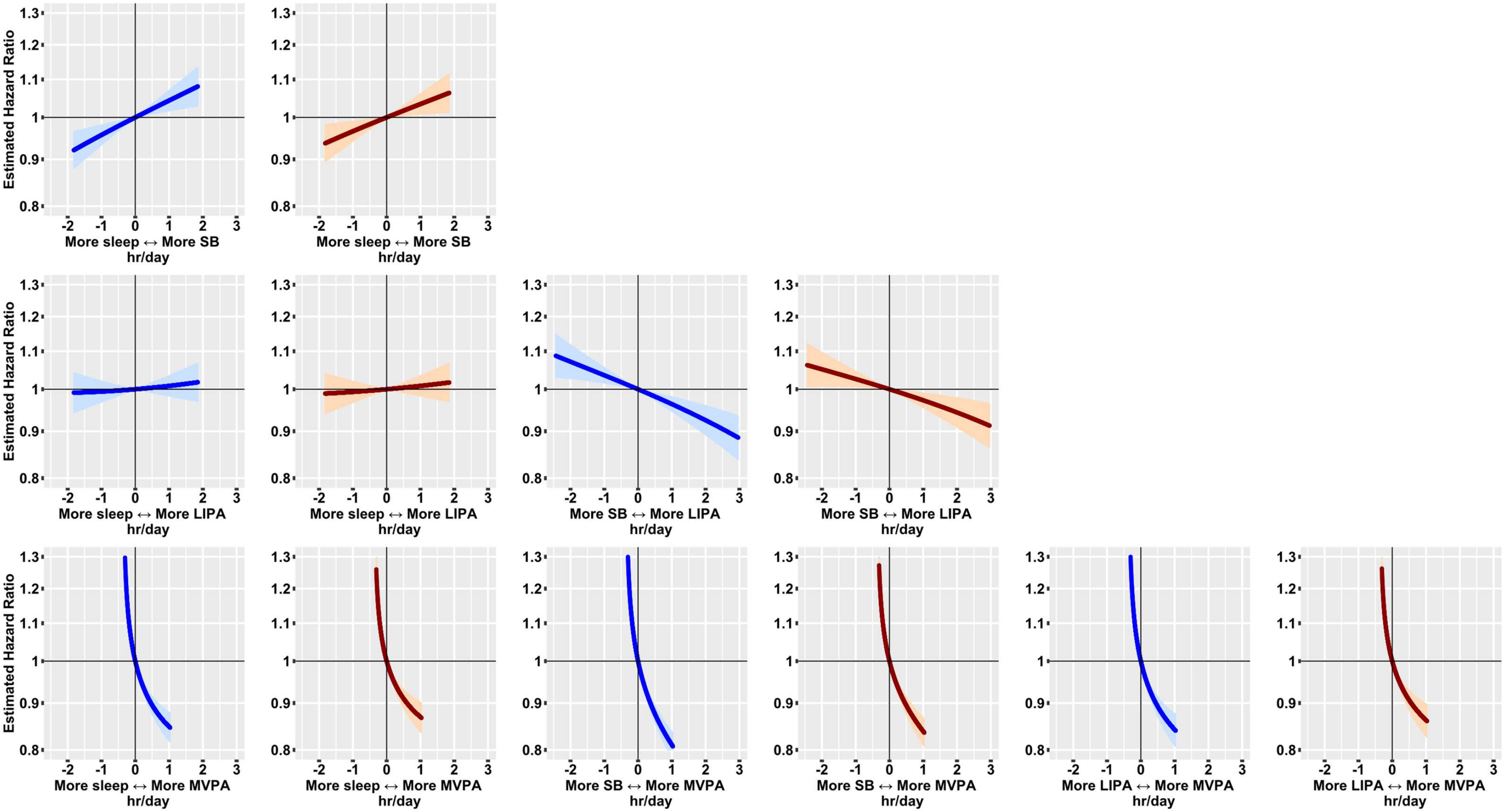

[a]Model based on 4,105 events in 87,498 participants. All relative to the mean behaviour composition: 8·8 hours/day sleep, 9·3 hours/day sedentary behaviour, 5·6 hours/day light physical activity behaviours, 0·35 hours/day (21 minutes/day) moderate-to-vigorous physical activity behaviours. Models used age as the timescale, were stratified by sex and were additionally adjusted for ethnicity, smoking status, alcohol consumption, fresh fruit and vegetable consumption, red and processed meat consumption, oily fish consumption, deprivation and education.

Supplementary Figure 5: Hazard Ratios for all (blue) and fatal (red) incident cardiovascular disease for all behaviour pairs estimated using a multivariable-adjusted Cox regression model.[a]

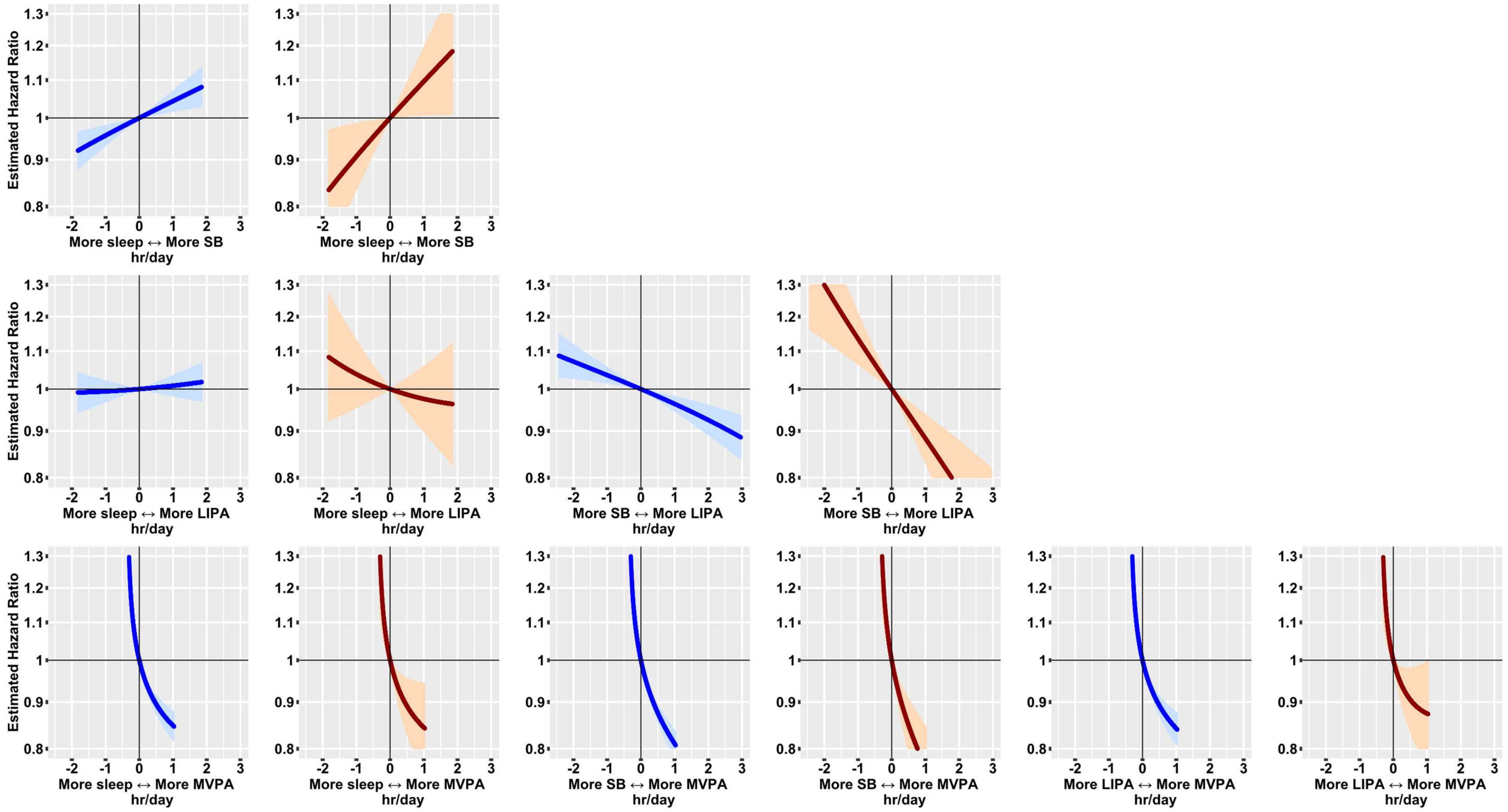

[a]Models based on 4,105 events and 348 cardiovascular deaths in 87,498 participants. All relative to the mean behaviour composition: 8·8 hours/day sleep, 9·3 hours/day sedentary behaviour, 5·6 hours/day light physical activity behaviours, 0·35 hours/day (21 minutes/day) moderate-to-vigorous physical activity behaviours. Models used age as the timescale, were stratified by sex and were additionally adjusted for ethnicity, smoking status, alcohol consumption, fresh fruit and vegetable consumption, red and processed meat consumption, oily fish consumption, deprivation and education.

Supplementary Figure 6: Hazard Ratios for incident cardiovascular disease for all behaviour pairs estimated using a multivariable-adjusted Cox regression model for women (blue) and men (red).[a]

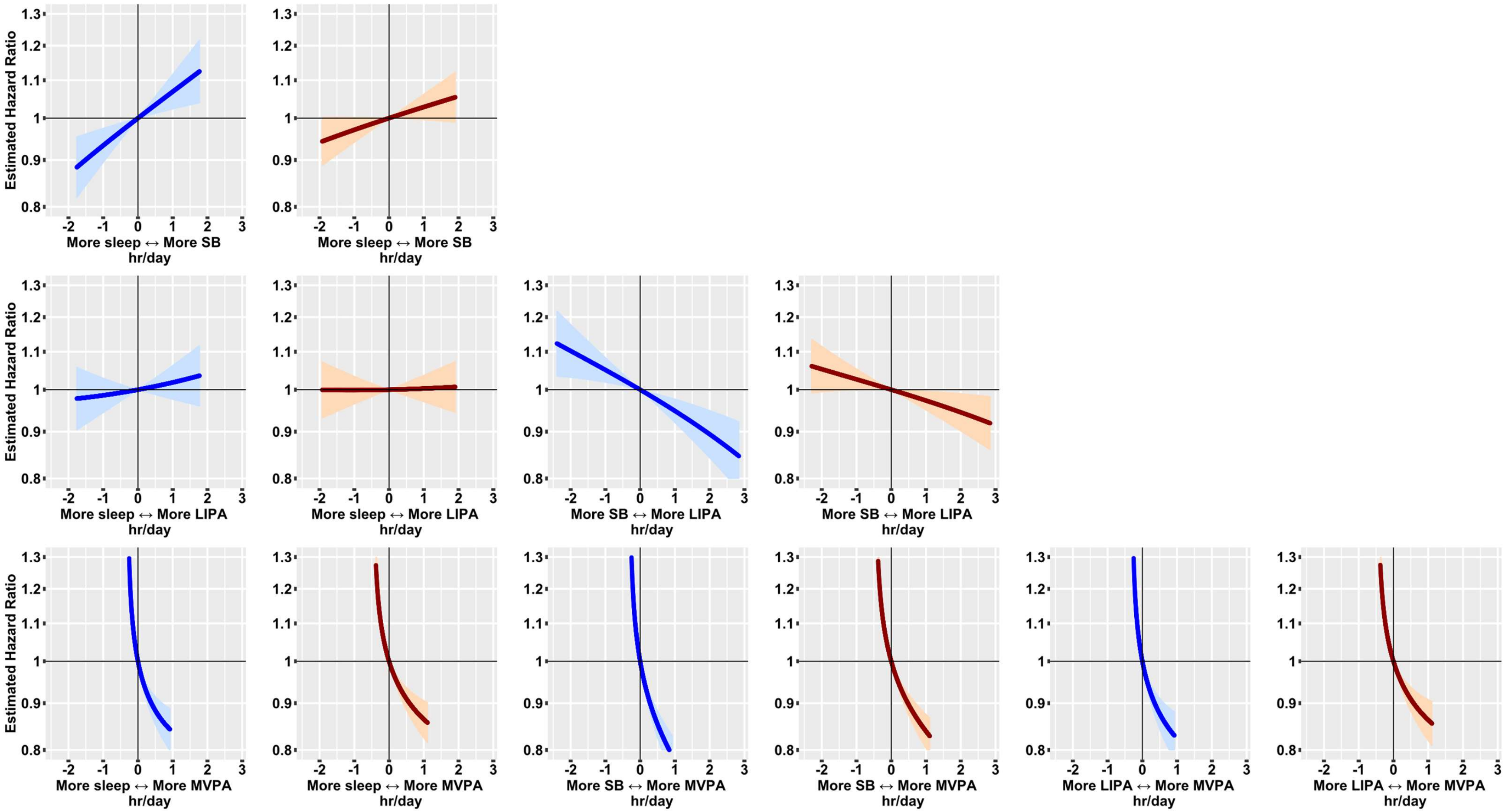

[a]Models based on 1,654 events in 50,882 women and 2,451 events in 36,616 men. All relative to the mean behaviour composition in each case (women – 8·9 hours/day sleep, 9·0 hours/day sedentary behaviour, 5·9 hours/day light physical activity behaviours, 0·30 hours/day (18 minutes/day) moderate-to-vigorous physical activity behaviours; men – 8·7 hours/day sleep, 9·7 hours/day sedentary behaviour, 5·1 hours/day light physical activity behaviours, 0·45 hours/day (27 minutes/day) moderate-to-vigorous physical activity behaviours). Models used age as the timescale and were additionally adjusted for ethnicity, smoking status, alcohol consumption, fresh fruit and vegetable consumption, red and processed meat consumption, oily fish consumption, deprivation and education.

Walmsley R, *et al. Br J Sports Med* 2022; 56:1008–1017. doi: 10.1136/bjsports-2021-104050

Supplementary Figure 7: Hazard Ratios for incident cardiovascular disease for all behaviour pairs estimated using a multivariable-adjusted Cox regression model for participants aged under 65 (blue) and participants aged over 65 (red).[a]

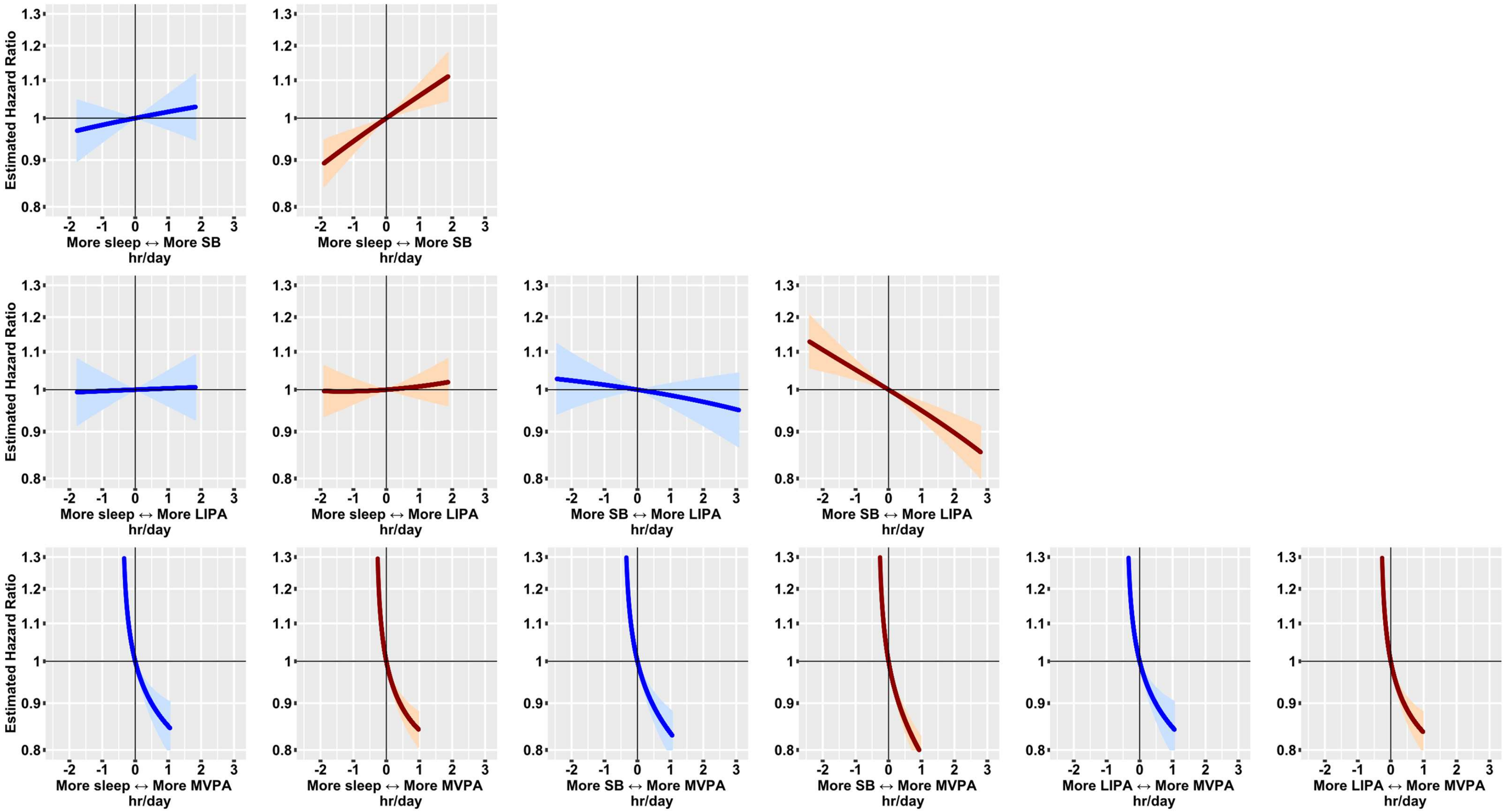

[a]Models based on 1,425 events in 51,180 participants aged under 65 and 2,680 events in 36,318 participants aged over 65. All relative to the mean behaviour composition in each case (participants aged under 65 – 8·8 hours/day sleep, 9·3 hours/day sedentary behaviour, 5·5 hours/day light physical activity behaviours, 0·39 hours/day (23 minutes/day) moderate-to-vigorous physical activity behaviours; participants aged over 65 – 8·9 hours/day sleep, 9·2 hours/day sedentary behaviour, 5·6 hours/day light physical activity behaviours, 0·30 hours/day (18 minutes/day) moderate-to-vigorous physical activity behaviours). Models used age as the timescale, were stratified by sex and were additionally adjusted for ethnicity, smoking status, alcohol consumption, fresh fruit and vegetable consumption, red and processed meat consumption, oily fish consumption, deprivation and education.

Walmsley R, *et al. Br J Sports Med* 2022; 56:1008–1017. doi: 10.1136/bjsports-2021-104050

Supplementary Figure 8: Hazard Ratios for incident cardiovascular disease for all behaviour pairs estimated using a multivariable-adjusted Cox regression model (blue), after removing the first two years of follow-up (red) and after additionally restricting to a healthy subgroup (green).[a]

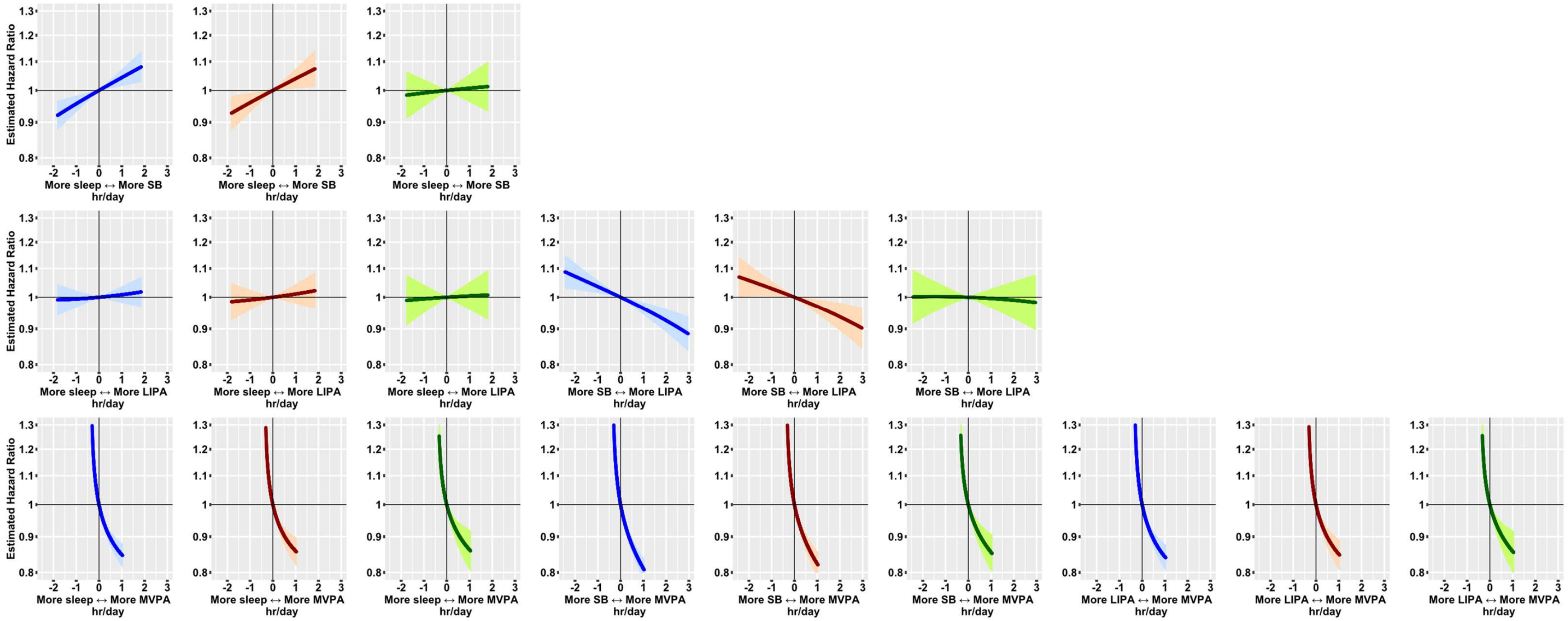

[a]Main analysis based on 4,105 events in 87,498 participants. First sensitivity analysis based on 2,947 events in 86,011 participants. Second sensitivity analysis based on 1,597 events in 63,267 participants. Models used age as the timescale, were stratified by sex and were additionally adjusted for ethnicity, smoking status, alcohol consumption, fresh fruit and vegetable consumption, red and processed meat consumption, oily fish consumption, deprivation and education. All values reported relative to the mean behaviour composition in each case:

Main analysis - 8·8 hours/day sleep, 9·3 hours/day sedentary behaviour, 5·6 hours/day light physical activity behaviours, 0·35 hours/day (21 minutes/day) moderate-to-vigorous physical activity behaviours

1st sensitivity - 8·8 hours/day sleep, 9·3 hours/day sedentary behaviour, 5·6 hours/day light physical activity behaviours, 0·35 hours/day (21 minutes/day) moderate-to-vigorous physical activity behaviours

2nd sensitivity - 8·8 hours/day sleep, 9·2 hours/day sedentary behaviour, 5·6 hours/day light physical activity behaviours, 0·39 hours/day (23 minutes/day) moderate-to-vigorous physical activity behaviours.

Walmsley R, *et al. Br J Sports Med* 2022; 56:1008–1017. doi: 10.1136/bjsports-2021-104050

Supplementary Figure 9: Hazard Ratios for cardiovascular disease (blue) and for non-activity-related accidents (red) for all behaviour pairs estimated using a multivariable-adjusted Cox regression model.[a]

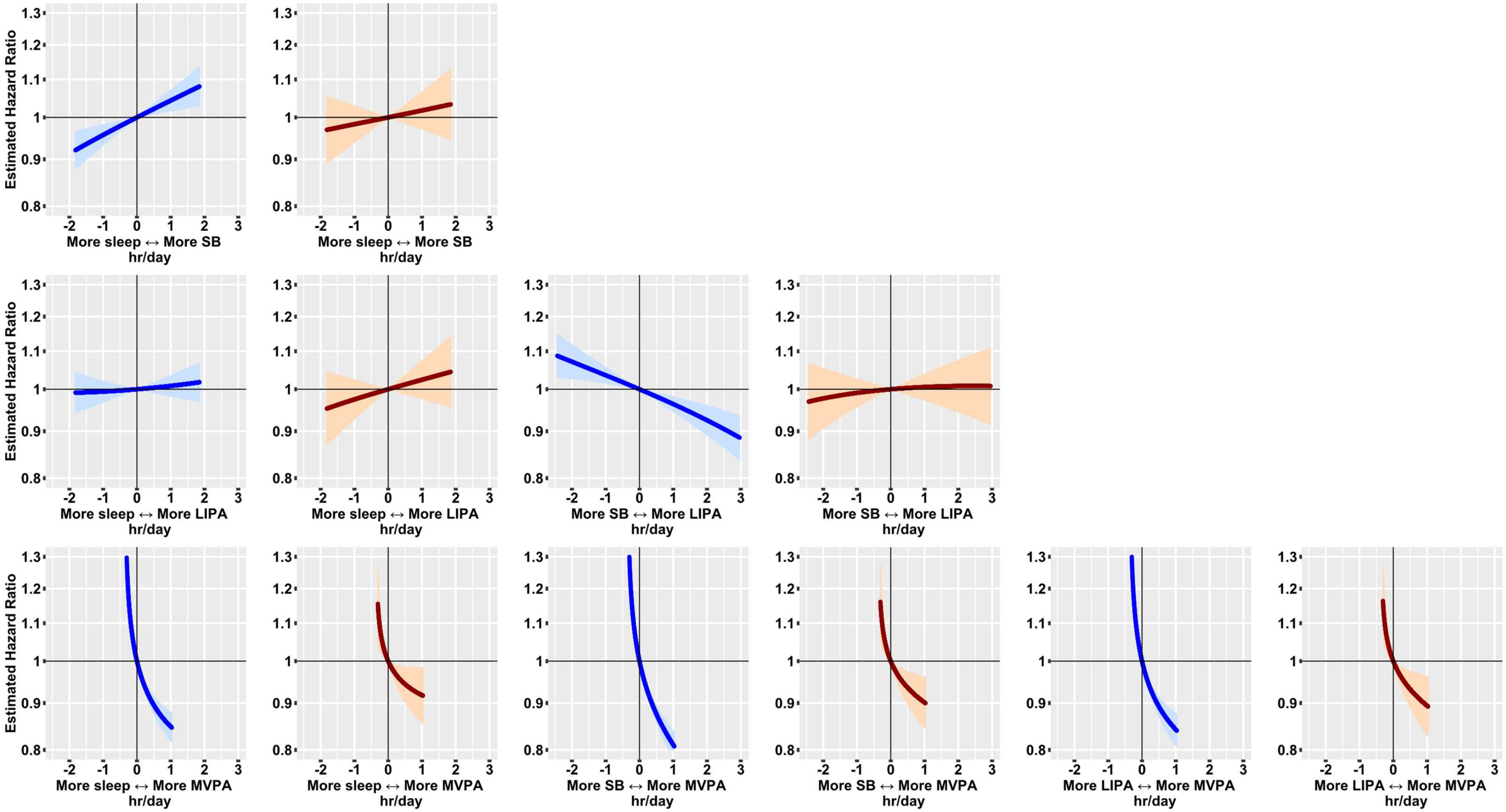

[a]Main model based on 4,105 events in 87,498 participants. Negative control model based on 1,375 events in 84,552 participants (participants with prior accident additionally excluded). All relative to the mean behaviour composition in each case (both 8·8 hours/day sleep, 9·3 hours/day sedentary behaviour, 5·6 hours/day light physical activity behaviours, 0·35 hours/day (21 minutes/day) moderate-to-vigorous physical activity behaviours). Models used age as the timescale, were stratified by sex and were additionally adjusted for ethnicity, smoking status, alcohol consumption, fresh fruit and vegetable consumption, red and processed meat consumption, oily fish consumption, deprivation and education.

Walmsley R, *et al. Br J Sports Med* 2022; 56:1008–1017. doi: 10.1136/bjsports-2021-104050

Supplementary Figure 10: Hazard ratios and corresponding E-values for incident cardiovascular disease associated with reallocating time to named behaviour, from all other behaviours proportionally, in 87,498 UK Biobank participants.[a]

	Hazard Ratio (95% CI)	E-value: Estimate (Interval bound)
REFERENCE: At compositional mean	1	
20 min/day more MVPA	0.91 (0.90, 0.93)	1.42 (1.36)
1 hr/day more LIPA	0.99 (0.98, 1.01)	1.09 (1.00)
1 hr/day more SB	1.05 (1.03, 1.07)	1.28 (1.20)
1 hr/day more sleep	0.98 (0.96, 1.00)	1.16 (1.00)

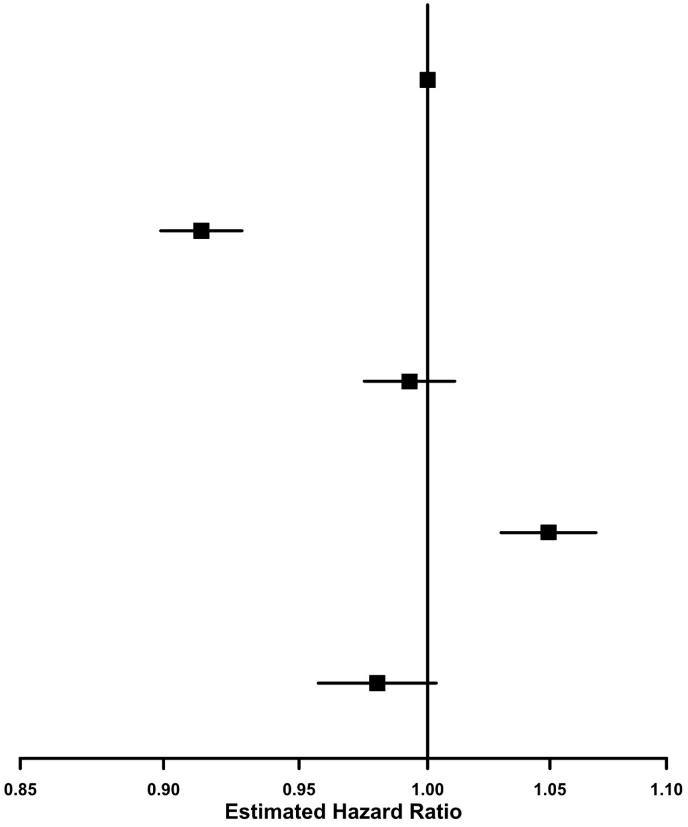

[a]Model based on 4,105 events in 87,498 participants. All relative to the mean behaviour composition (8·8 hours/day sleep, 9·3 hours/day sedentary behaviour, 5·6 hours/day light physical activity behaviours, 0·35 hours/day (21 minutes/day) moderate-to-vigorous physical activity behaviours) and more time in named behaviour reallocated from all other behaviours proportionally. Model used age as the timescale, was stratified by sex and was additionally adjusted for ethnicity, smoking status, alcohol consumption, fresh fruit and vegetable consumption, red and processed meat consumption, oily fish consumption, deprivation and education.

Walmsley R, *et al. Br J Sports Med* 2022; 56:1008–1017. doi: 10.1136/bjsports-2021-104050

Supplementary Figure 11: An example of a decision tree to classify time windows using average acceleration vector magnitude (avm) and the 75th percentile of acceleration vector magnitude (75thp).

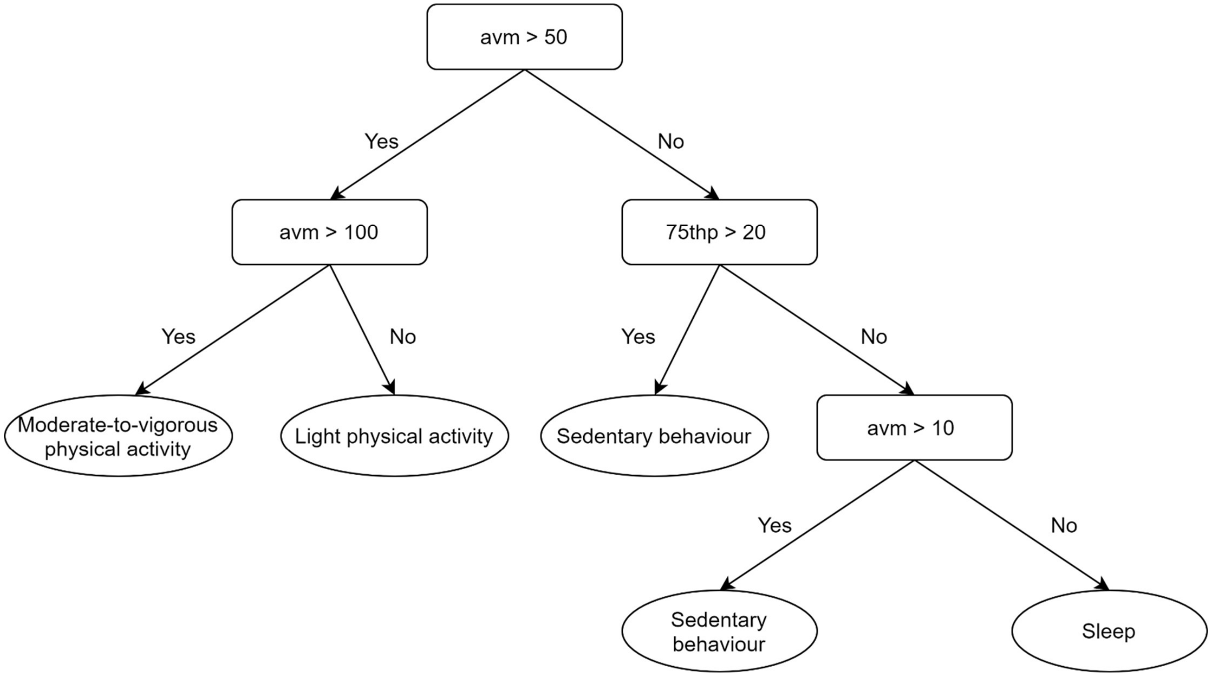

Walmsley R, *et al. Br J Sports Med* 2022; 56:1008–1017. doi: 10.1136/bjsports-2021-104050

Supplementary Figure 12: The structure of a Hidden Markov Model.

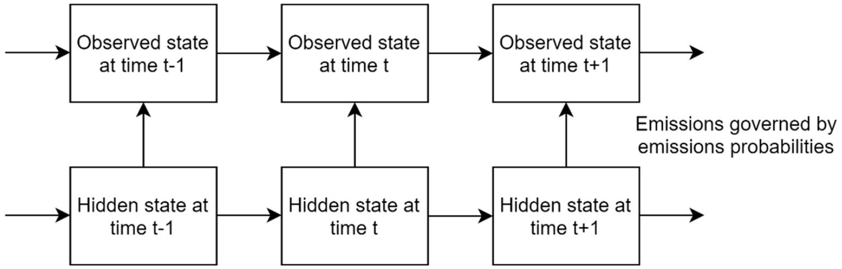

Walmsley R, *et al. Br J Sports Med* 2022; 56:1008–1017. doi: 10.1136/bjsports-2021-104050

Supplementary Figure 13: Hazard Ratios for incident cardiovascular disease for all behaviour pairs estimated using a multivariable-adjusted Cox regression model for all participants (blue) and in a sensitivity analysis excluding individuals with a zero value in any behaviour (red).[a]

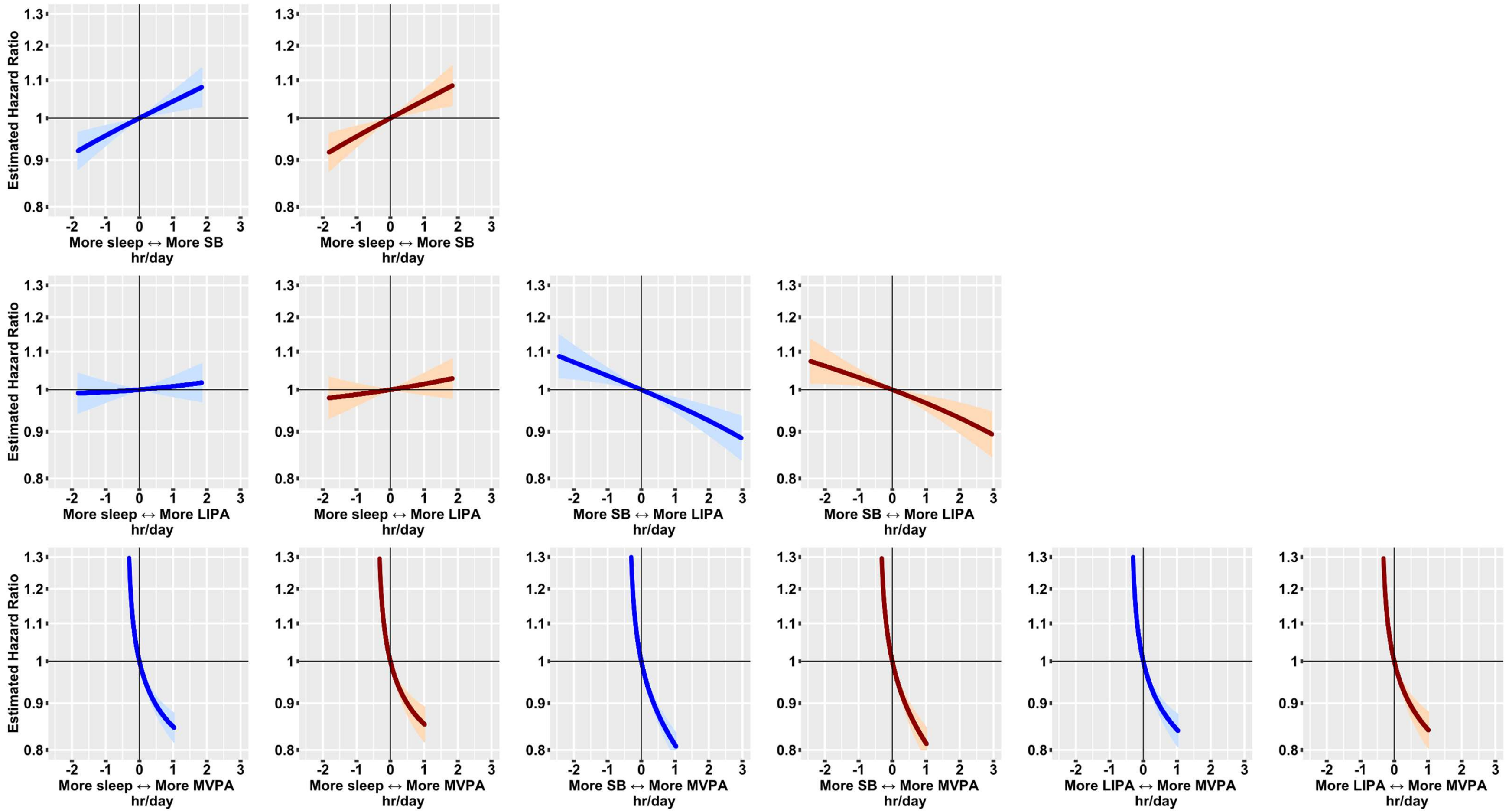

[a]Main model based on 4,105 events in 87,498 participants. Model excluding individuals with zero values based on 4,017 events in 86,696 participants. All relative to the mean behaviour composition in each case (main analysis – 8·8 hours/day sleep, 9·3 hours/day sedentary behaviour, 5·6 hours/day light physical activity behaviours, 0·35 hours/day (21 minutes/day) moderate-to-vigorous physical activity behaviours; analysis excluding individuals with zero values – 8·8 hours/day sleep, 9·3 hours/day sedentary behaviour, 5·6 hours/day light physical activity behaviours, 0·37 hours/day (22 minutes/day) moderate-to-vigorous physical activity behaviours). Models used age as the timescale, were stratified by sex and were additionally adjusted for ethnicity, smoking status, alcohol consumption, fresh fruit and vegetable consumption, red and processed meat consumption, oily fish consumption, deprivation and education.

Supplementary Figure 14: Hazard Ratios for incident cardiovascular disease estimated using a multivariable-adjusted Compositional Data Analysis Cox regression model (blue) and using a multivariable-adjusted linear isotemporal substitution Cox regression model (red).[a]

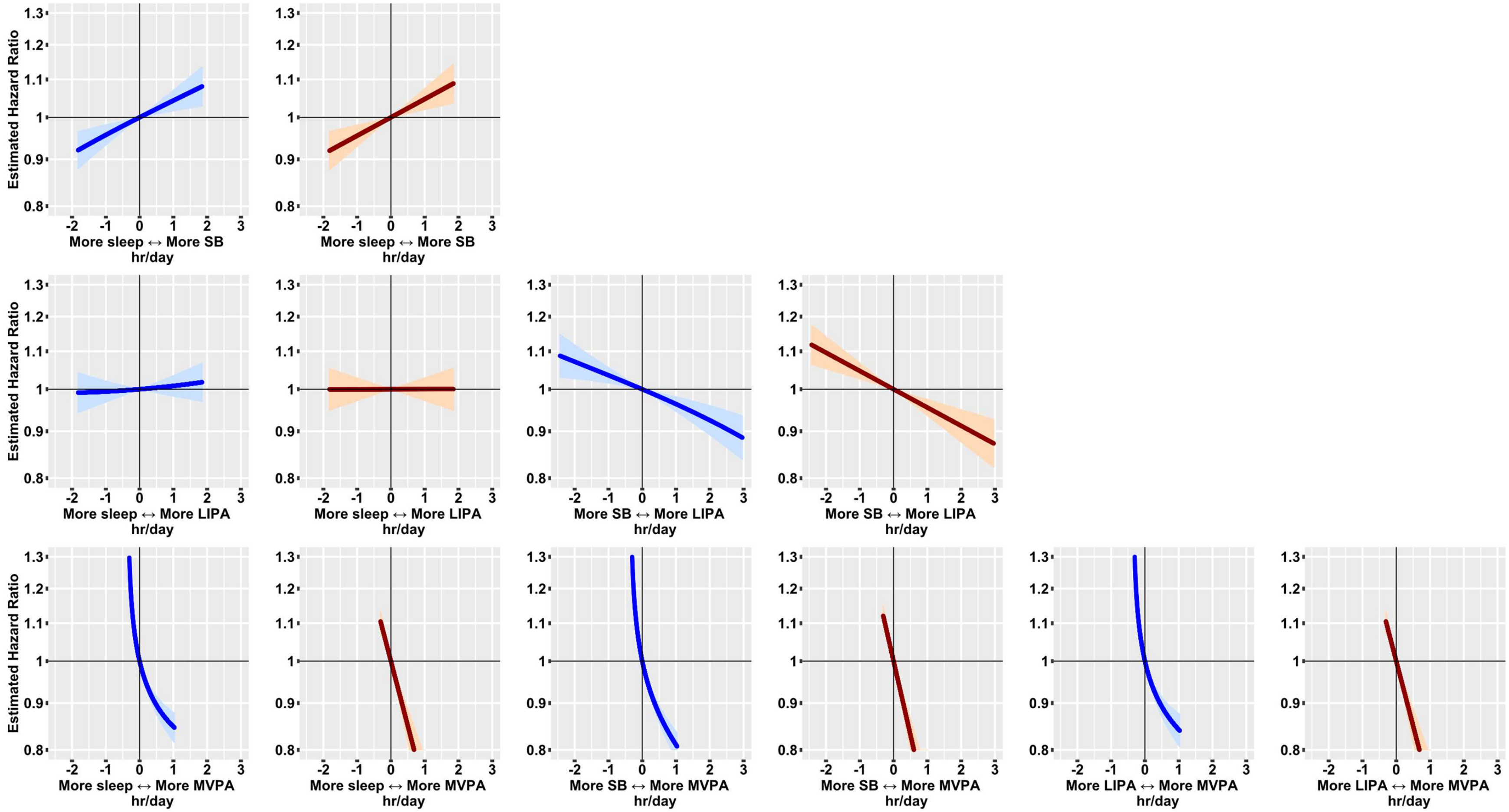

[a]Model based on 4,105 events in 87,498 participants. All relative to the mean behaviour composition: 8·8 hours/day sleep, 9·3 hours/day sedentary behaviour, 5·6 hours/day light physical activity behaviours, 0·35 hours/day (21 minutes/day) moderate-to-vigorous physical activity behaviours. Models used age as the timescale, were stratified by sex and were additionally adjusted for ethnicity, smoking status, alcohol consumption, fresh fruit and vegetable consumption, red and processed meat consumption, oily fish consumption, deprivation and education.

Walmsley R, *et al. Br J Sports Med* 2022; 56:1008–1017. doi: 10.1136/bjsports-2021-104050

STROBE Statement—Checklist of items that should be included in reports of cohort studies

	Item No	Recommendation	Included
Title and abstract	1	(*a*) Indicate the study's design with a commonly used term in the title or the abstract	Yes
		(*b*) Provide in the abstract an informative and balanced summary of what was done and what was found	Yes
**Introduction**			
Background/rationale	2	Explain the scientific background and rationale for the investigation being reported	Yes
Objectives	3	State specific objectives, including any prespecified hypotheses	Yes, No pre-specified hypotheses
**Methods**			
Study design	4	Present key elements of study design early in the paper	Yes
Setting	5	Describe the setting, locations, and relevant dates, including periods of recruitment, exposure, follow-up, and data collection	Yes
Participants	6	(*a*) Give the eligibility criteria, and the sources and methods of selection of participants. Describe methods of follow-up	Yes
		(*b*) For matched studies, give matching criteria and number of exposed and unexposed	NA
Variables	7	Clearly define all outcomes, exposures, predictors, potential confounders, and effect modifiers. Give diagnostic criteria, if applicable	Yes
Data sources/ measurement	8*	For each variable of interest, give sources of data and details of methods of assessment (measurement). Describe comparability of assessment methods if there is more than one group	Yes
Bias	9	Describe any efforts to address potential sources of bias	Yes
Study size	10	Explain how the study size was arrived at	Yes
Quantitative variables	11	Explain how quantitative variables were handled in the analyses. If applicable, describe which groupings were chosen and why	Yes
Statistical methods	12	(*a*) Describe all statistical methods, including those used to control for confounding	Yes
		(*b*) Describe any methods used to examine subgroups and interactions	Yes
		(*c*) Explain how missing data were addressed	Yes
		(*d*) If applicable, explain how loss to follow-up was addressed	Yes
		(*e*) Describe any sensitivity analyses	Yes

Results

Participants	13*	(a) Report numbers of individuals at each stage of study—eg numbers potentially eligible, examined for eligibility, confirmed eligible, included in the study, completing follow-up, and analysed	Yes
		(b) Give reasons for non-participation at each stage	Yes
		(c) Consider use of a flow diagram	Yes
Descriptive data	14*	(a) Give characteristics of study participants (eg demographic, clinical, social) and information on exposures and potential confounders	Yes
		(b) Indicate number of participants with missing data for each variable of interest	Yes
		(c) Summarise follow-up time (eg, average and total amount)	Yes
Outcome data	15*	Report numbers of outcome events or summary measures over time	Yes
Main results	16	(*a*) Give unadjusted estimates and, if applicable, confounder-adjusted estimates and their precision (eg, 95% confidence interval). Make clear which confounders were adjusted for and why they were included	Yes: confounder-adjusted estimates and estimates adjusted for only age and sex given.
		(*b*) Report category boundaries when continuous variables were categorized	Yes.
		(*c*) If relevant, consider translating estimates of relative risk into absolute risk for a meaningful time period	Not included.
Other analyses	17	Report other analyses done—eg analyses of subgroups and interactions, and sensitivity analyses	Yes.
**Discussion**			
Key results	18	Summarise key results with reference to study objectives	Yes.
Limitations	19	Discuss limitations of the study, taking into account sources of potential bias or imprecision. Discuss both direction and magnitude of any potential bias	Yes.
Interpretation	20	Give a cautious overall interpretation of results considering objectives, limitations, multiplicity of analyses, results from similar studies, and other relevant evidence	Yes.
Generalisability	21	Discuss the generalisability (external validity) of the study results	Yes.
**Other information**			
Funding	22	Give the source of funding and the role of the funders for the present study and, if applicable, for the original study on which the present article is based	Yes.

*Give information separately for exposed and unexposed groups.

Note: An Explanation and Elaboration article discusses each checklist item and gives methodological background and published examples of transparent reporting. The STROBE checklist is best used in conjunction with this article (freely available on the Web sites of PLoS Medicine at http://www.plosmedicine.org/, Annals of Internal Medicine at http://www.annals.org/, and Epidemiology at http://www.epidem.com/). Information on the STROBE Initiative is available at http://www.strobe-statement.org.
